# Supplementary material for: Biology-guided deep learning predicts prognosis and cancer immunotherapy response
Source: Nat Commun. 2023 Aug 23;14:5135. doi: 10.1038/s41467-023-40890-x (PMC10447467; doi:10.1038/s41467-023-40890-x)
Supplement: Supplementary file 1 — Supplementary Information [file 41467_2023_40890_MOESM1_ESM.pdf]

Supplementary Information

- **Supplementary Figures**
- **Supplementary Tables**

Supplementary Figures

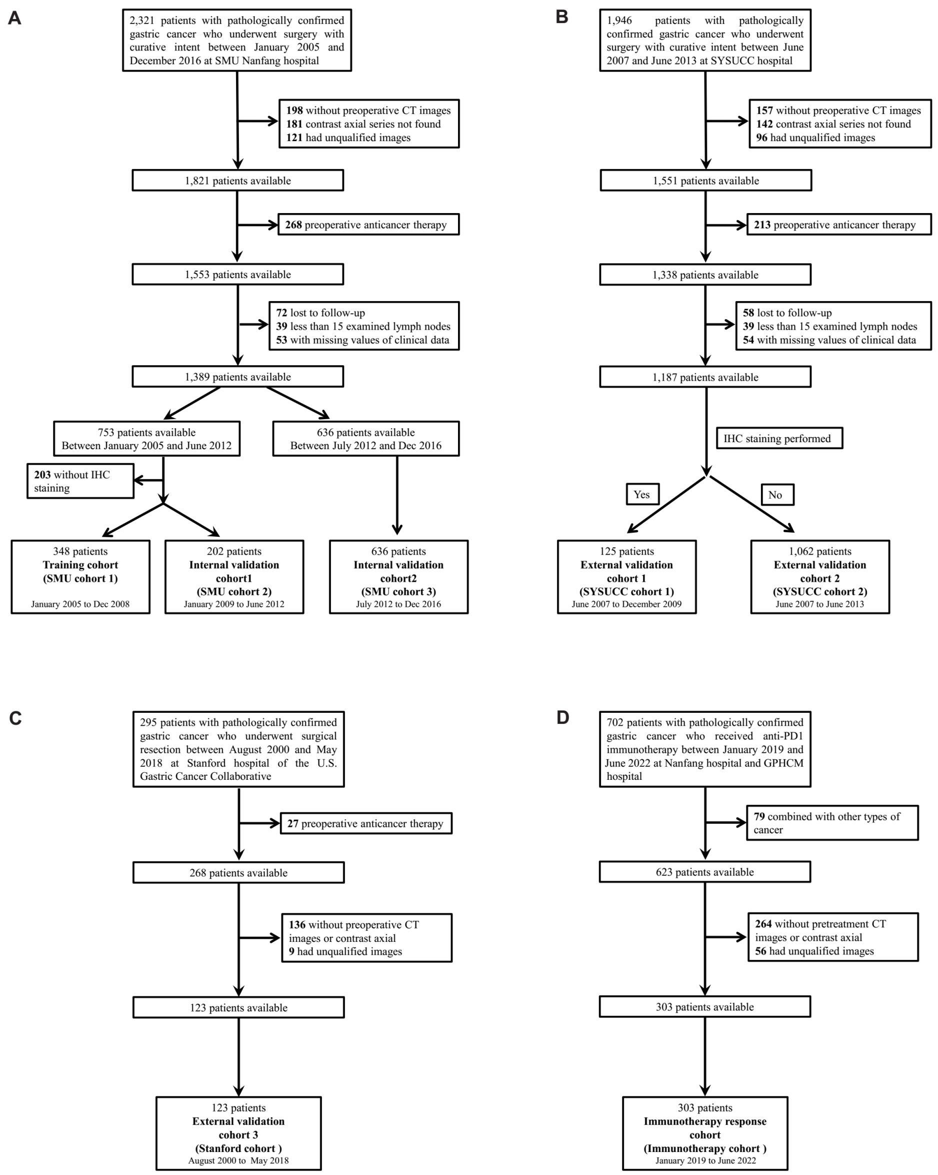

Supplementary Figure 1. Flow chart of patient inclusion and exclusion

(A) Patient recruitments of training cohort, internal validation cohort 1, and internal validation cohort 2. (B) Patient recruitments of external validation cohort 1 and external validation cohort 2. (C) Patient recruitment of external validation cohort 3. (D) Patient recruitment of immunotherapy response cohort. SMU cohorts: patients from Southern Medical University hospital; SYSUCC cohorts: patients from Sun Yat-sen University Cancer Center; Stanford cohort: patients from Stanford University hospital. Immunotherapy cohort: patients from Guangdong Provincial Hospital of Chinese Medicine (GPHCM).

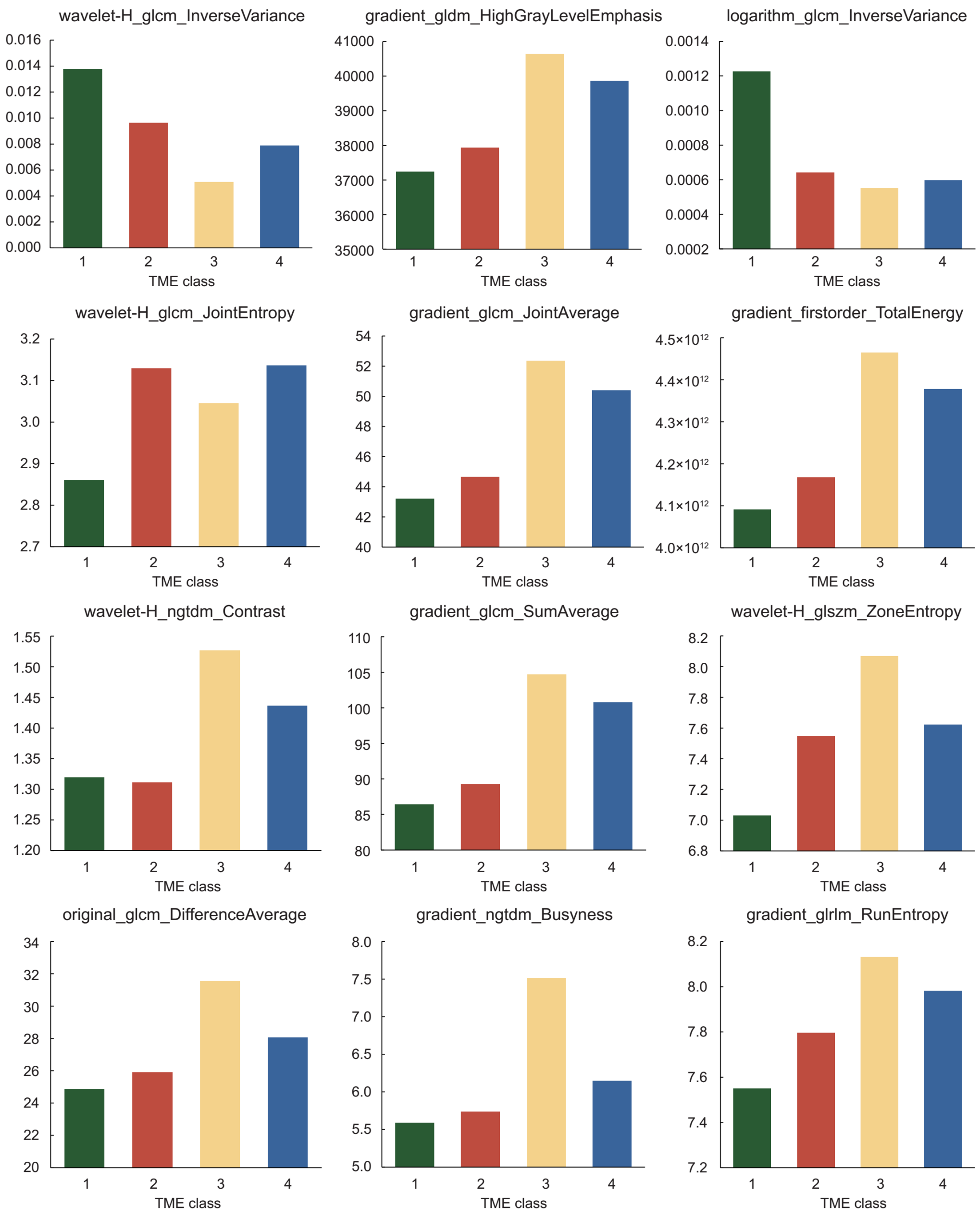

Supplementary Figure 2. Texture feature values of feature maps in different TME classes. The radiomics features are calculated based on the feature maps (15th channel) for each of the predicted TME classes. Features that measure heterogeneity generally show increasing patterns from TME class 1 through 4, while those measuring homogeneity (GLCM_InverseVariance) show decreasing patterns.

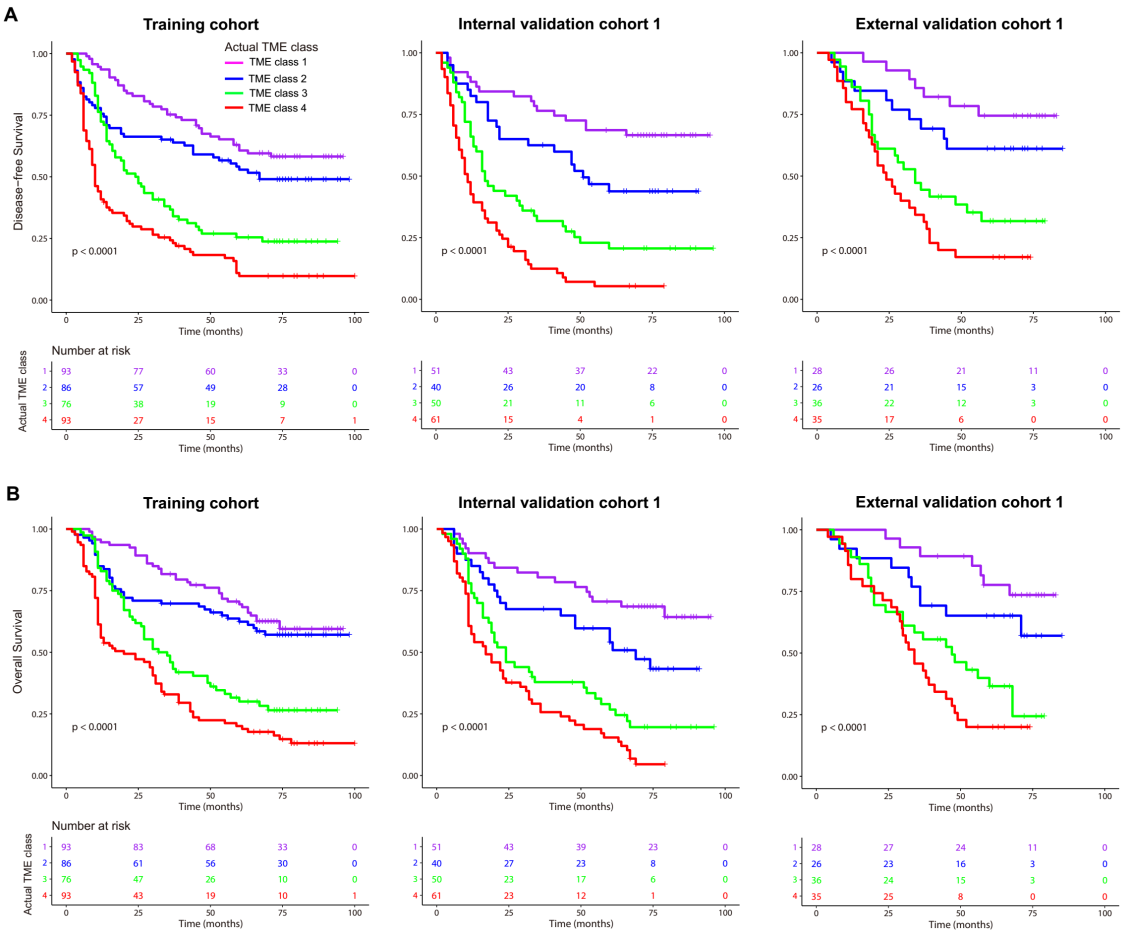

Supplementary Figure 3. Kaplan-Meier analyses of disease-free survival (DFS) and overall survival (OS) according to IHC-defined TME class in patients with gastric cancer. (A): disease-free survival (DFS) (B): overall survival (OS). Training cohort (SMU-1 cohort; n=348), Internal validation cohort 1 (SMU-2 cohort; n=202), External validation cohort 1 (SYSUCC-1 cohort; n=125). Comparisons of the survival curves were performed with a two-sided log-rank test. Source data are provided as a Source Data file.

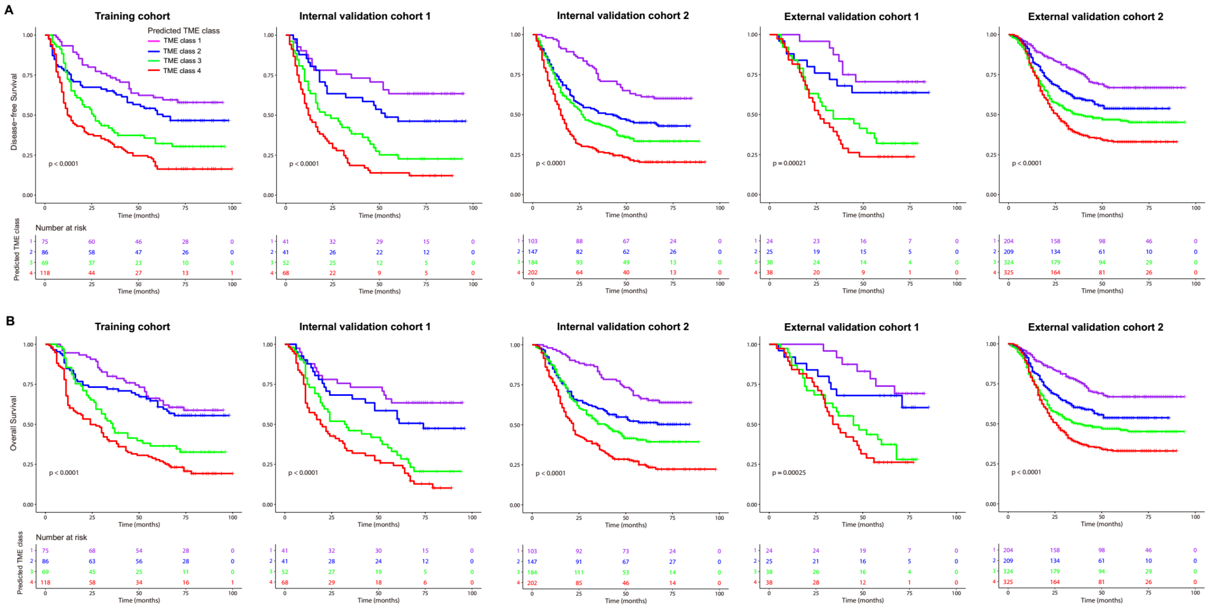

Supplementary Figure 4. Kaplan-Meier analyses of disease-free survival (DFS) and overall survival (OS) according to the CT image-based predicted TME class in patients with gastric cancer. (A): disease-free survival (DFS) (B): overall survival (OS). Training cohort (SMU-1 cohort; n=348), Internal validation cohort 1 (SMU-2 cohort; n=202), Internal validation cohort 2 (SMU-3 cohort; n=636), External validation cohort 1 (SYSUCC-1 cohort; n=125), External validation cohort 2 (SYSUCC-2 cohort; n=1062). Comparisons of the survival curves were performed with a two-sided log-rank test. Source data are provided as a Source Data file.

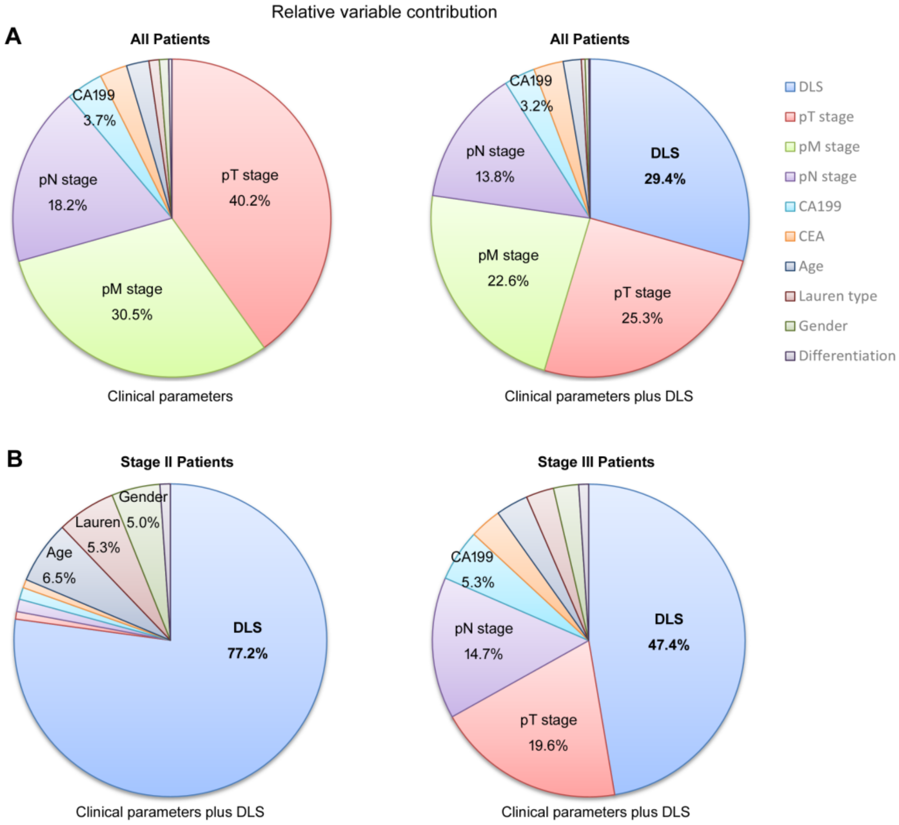

Supplementary Figure 5. Relative importance of each risk parameter to overall survival (OS) risk using the χ^2 proportion test (n=2025). A, clinical parameters (left) and clinical parameters plus DLS (right). B, stage II (left) and stage III (right). Source data are provided as a Source Data file.

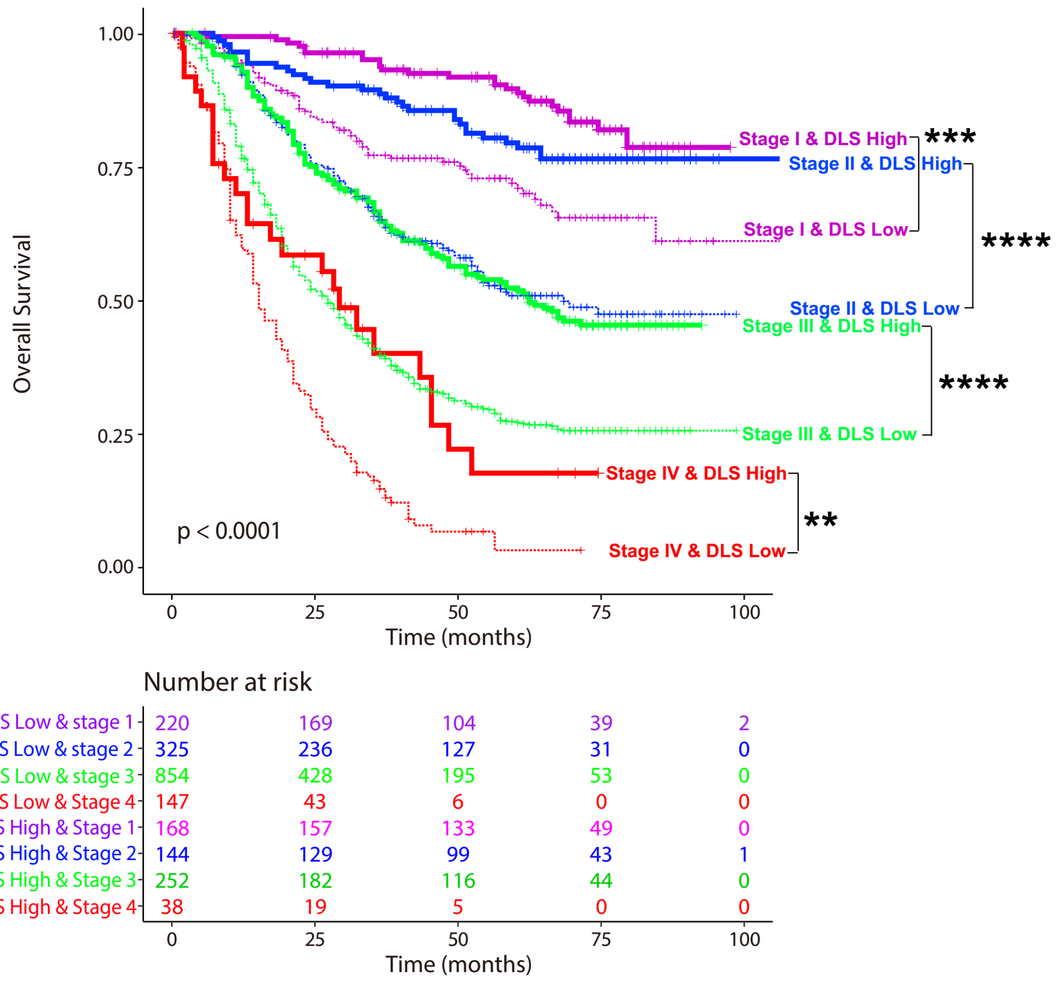

Supplementary Figure 6. Kaplan-Meier survival analysis of overall survival according to the predicted survival score within each stage in all the combined validation cohorts (n=2148).

** *P*<0.01, *** *P*<0.001. Comparisons of the survival curves were performed with a two-sided log-rank test. Source data are provided as a Source Data file.

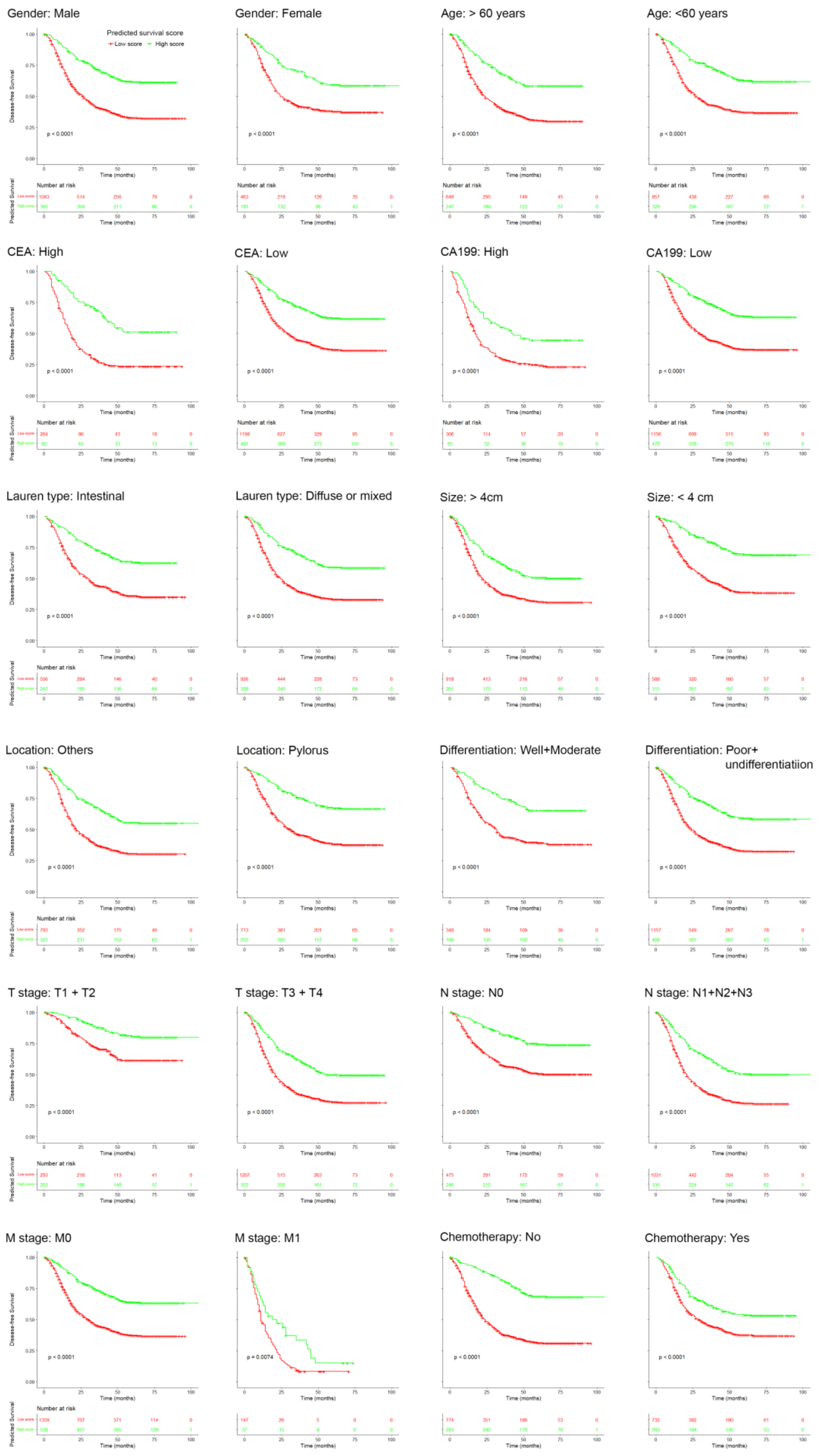

Supplementary Figure 7. Kaplan-Meier survival analysis of DFS for all the patients (n=2496) according to the predicted survival score stratified by clinicopathological risk factors. Comparisons of the survival curves were performed with a two-sided log-rank test. Source data are provided as a Source Data file.

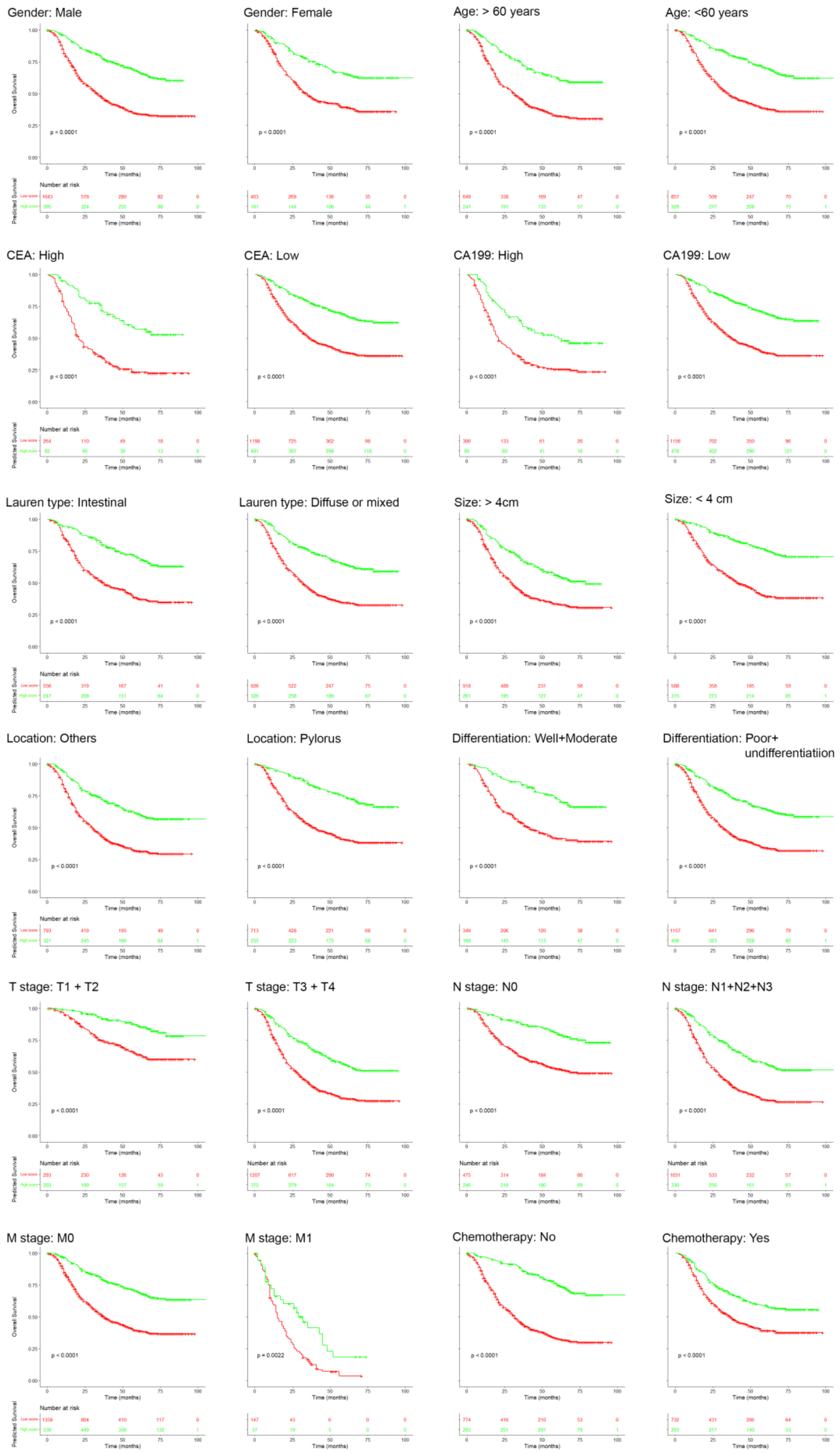

Supplementary Figure 8. Kaplan-Meier survival analysis of OS for all the patients (n=2496) according to the predicted survival score stratified by clinicopathological risk factors. Comparisons of the survival curves were performed with a two-sided log-rank test. Source data are provided as a Source Data file.

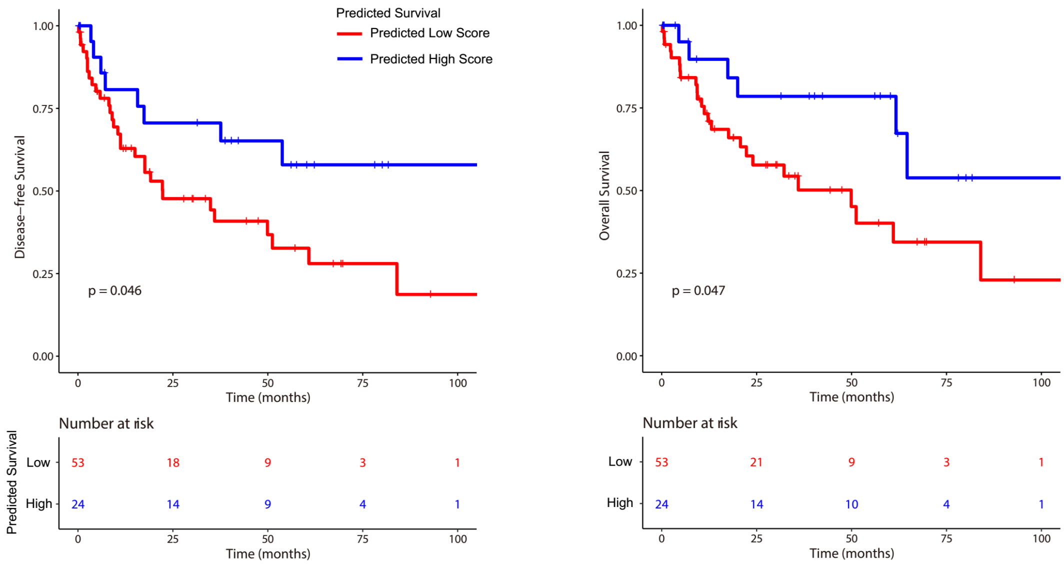

Supplementary Figure 9. Kaplan-Meier survival analysis of DFS and OS for the Non-Asian patients from Stanford cohort (n=77) according to the predicted survival score. Comparisons of the survival curves were performed with a two-sided log-rank test. Source data are provided as a Source Data file.

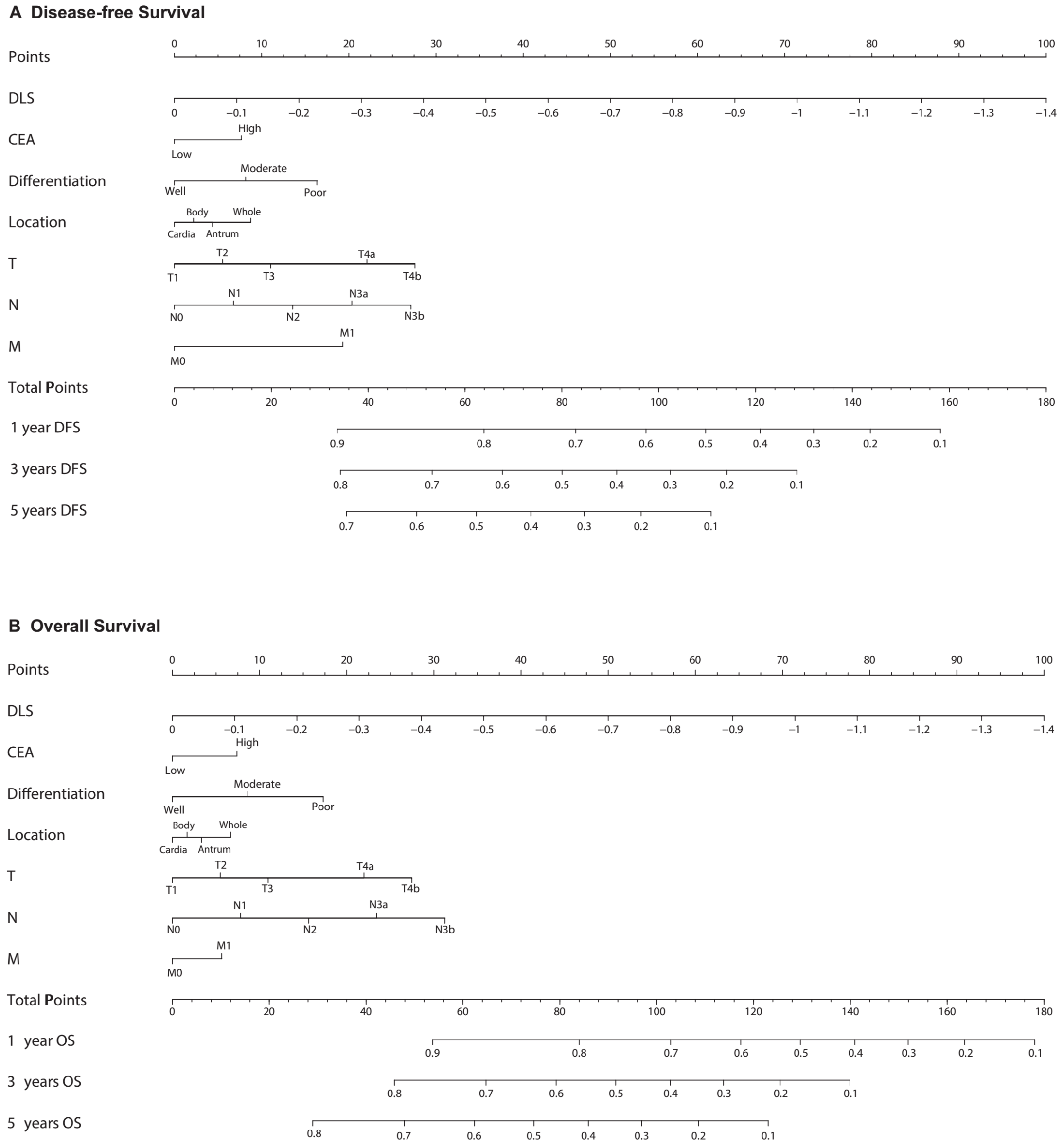

Supplementary Figure 10. Integrated nomograms to predict 1-, 3-, 5- year DFS and OS for patients with gastric cancer (n=348).

To determine how many points toward the probability of (A) DFS and (B) OS the patient receives for his or her predicted survival score, locate the patient's predicted survival score on the predicted survival score axis, draw a line straight upward to the point axis, repeat this process for each variable, sum the points achieved for each of the risk factors, locate the final sum on the Total Point axis, and draw a line straight down to find the patient's probability of DFS and OS. DFS: disease-free survival; OS: overall survival. Source data are provided as a Source Data file.

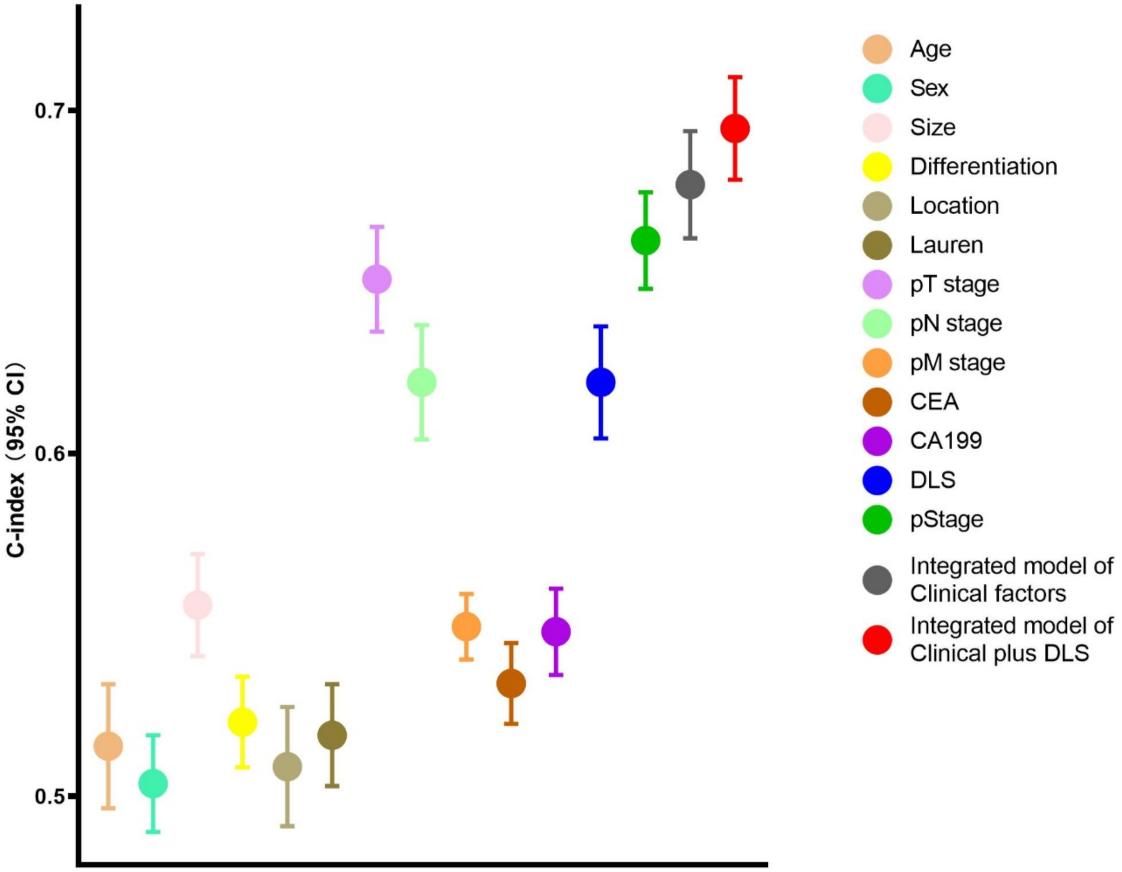

Supplementary Figure 11. **C-index of prediction for disease-free survival using clinicopathologic variable and the deep learning model.** n=2025. C-index: concordance index. The dot and error bar represent the mean and the 95% confidence intervals. Source data are provided as a Source Data file.

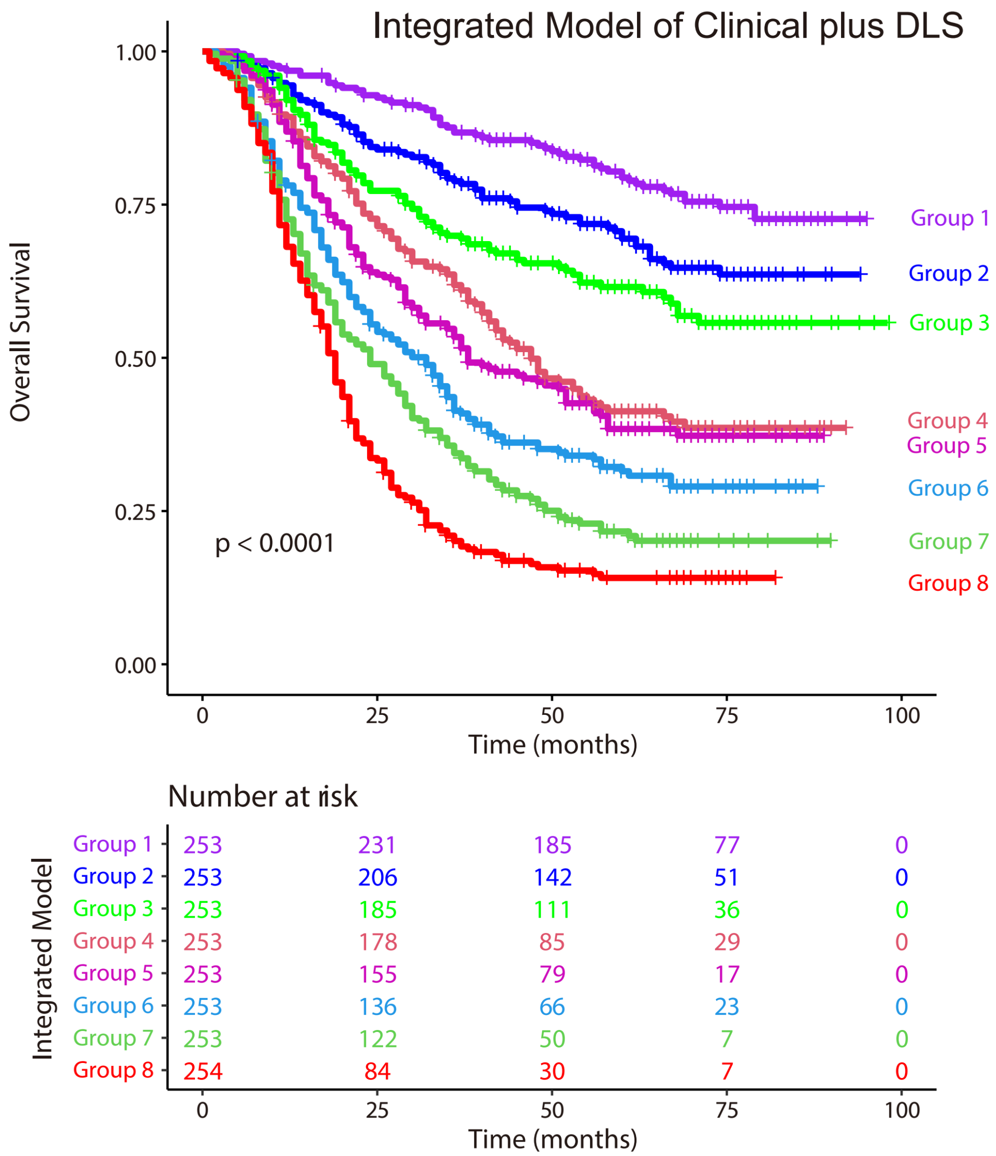

Supplementary Figure 12. Kaplan-Meier survival analysis of overall survival according to the integrated nomogram in the validation cohorts. Comparisons of the survival curves were performed with a two-sided log-rank test. n=2025. Source data are provided as a Source Data file.

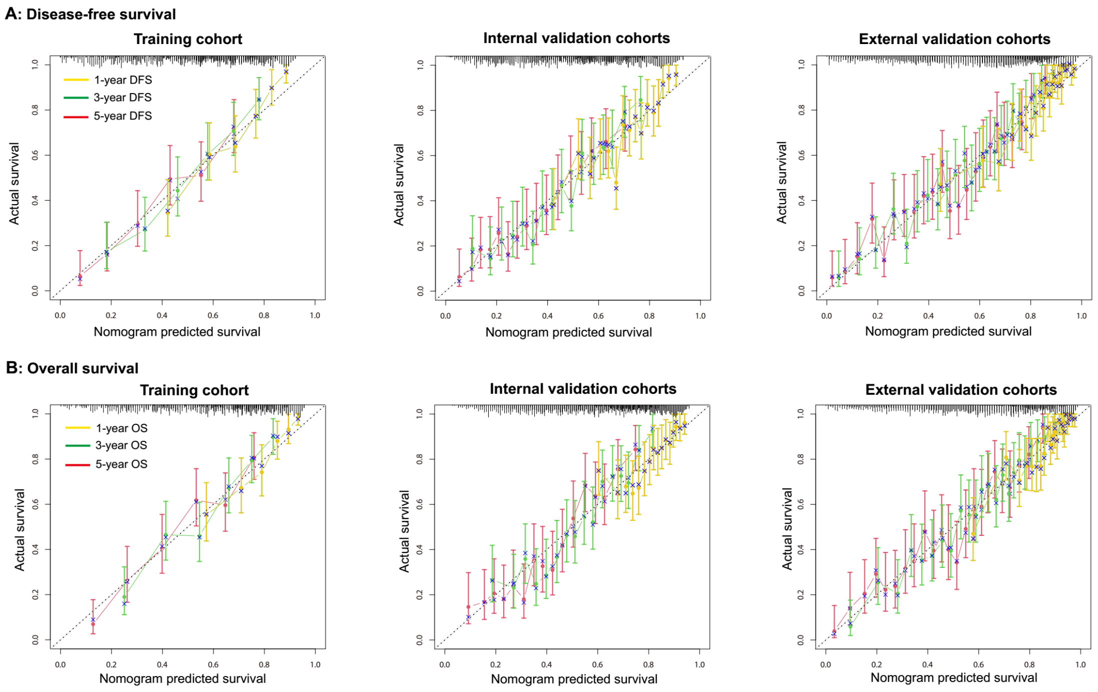

Supplementary Figure 13. Calibration curves for the integrated nomograms.

DFS (A) and OS (B). The calibration showed the agreement between the estimated and the observed 1-, 3-, and 5-year outcomes. Nomogram-estimated DFS or OS is plotted on the x-axis; the observed DFS or OS is plotted on the y-axis. The diagonal dotted line is a perfect estimation by an ideal model, in which the estimated outcome perfectly corresponds to the actual outcome. The solid line is the performance of the nomogram: a closer alignment with the diagonal dotted line represents a better estimation. Training cohort (n=348), internal validation cohort (n=838), external validation cohort (n=1310). The dot and error bar represent the mean and the 95% confidence intervals. Source data are provided as a Source Data file.

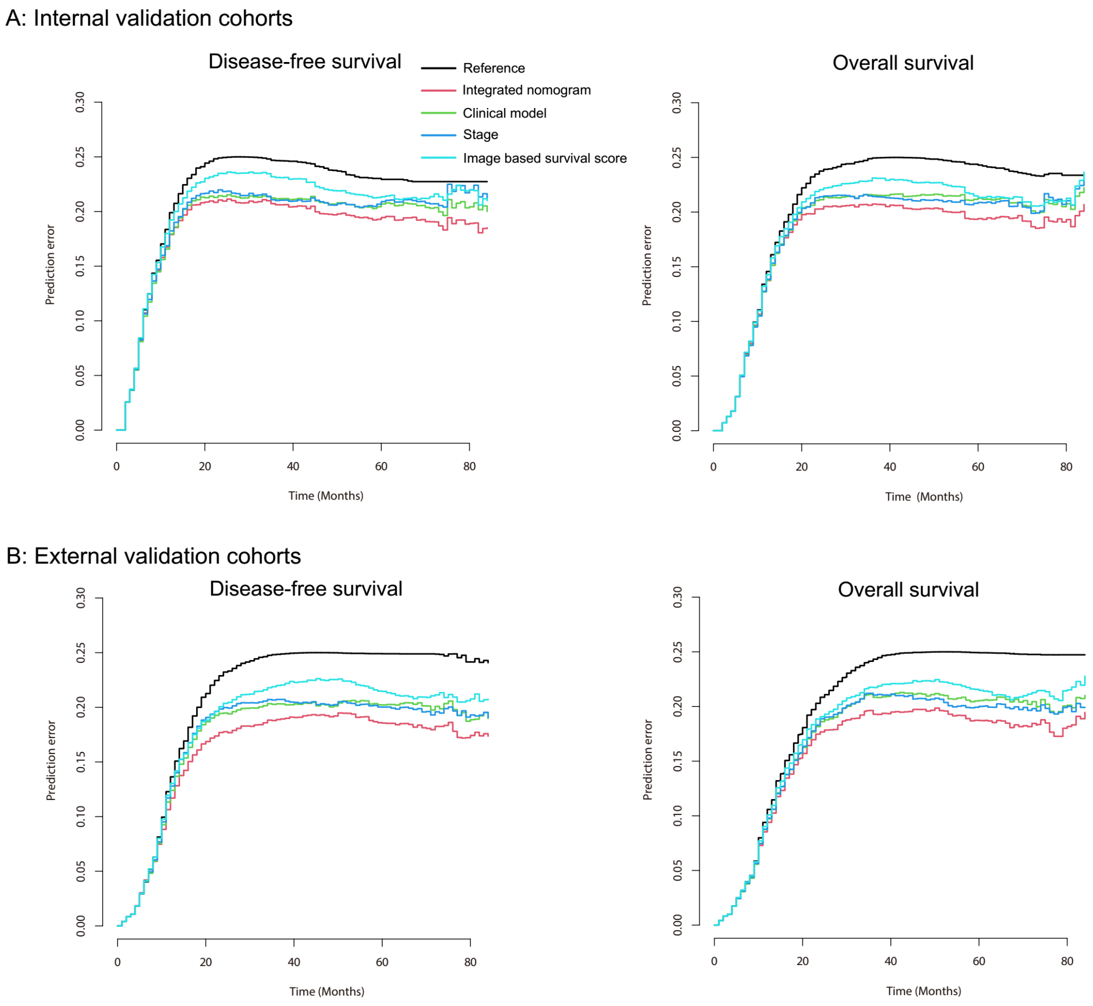

Supplementary Figure 14. Prediction error curves for each model in the study for stratifying disease-free survival and overall survival in the validation cohorts (n = 2025).

(A) Prediction error curves for each model in the study for stratifying disease-free survival and overall survival in the internal validation cohorts. (B) Prediction error curves for each model in the study for stratifying disease-free survival and overall survival in the external validation cohorts. Left panel: Disease-free survival; Right panel: Overall survival. Prediction error curves for the training cohort and validation cohorts (lower prediction errors indicate higher model accuracy). Source data are provided as a Source Data file.

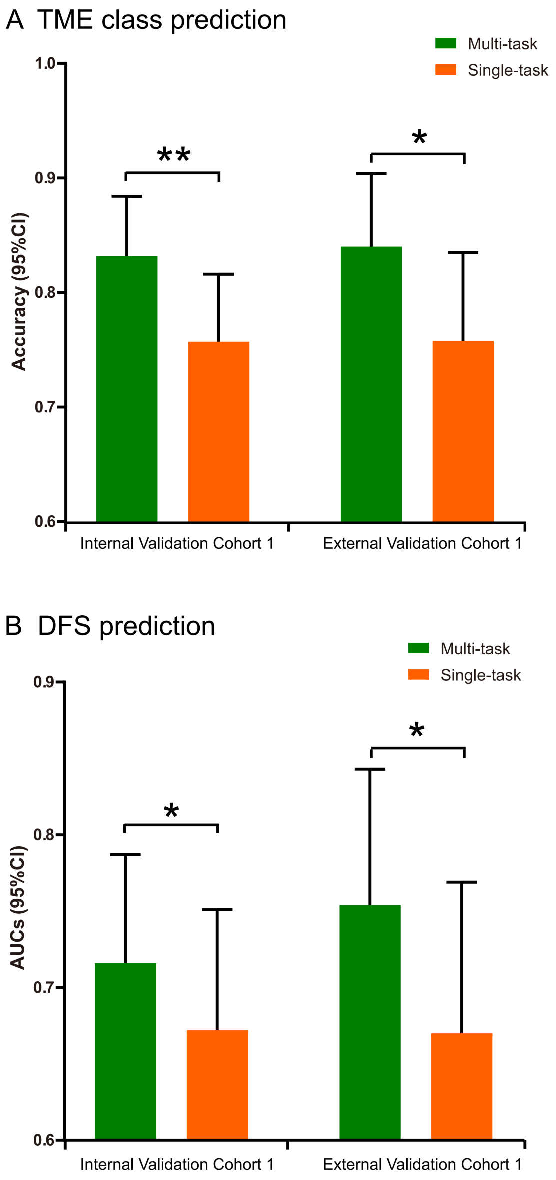

Supplementary Figure 15. Comparison of the multi-task learning approach with the single-task learning for prediction of TME class and disease-free survival.

(A) Accuracies of different approaches for prediction of TME class. (B) The AUCs of different approaches for prediction of disease-free survival. AUC: area under the ROC curves, TME：tumor microenvironment, DFS: disease-free survival. Internal validation cohort 1 (n=202), External validation cohort 1 (n=125). Comparisons of the bar plot were performed with a two-sided t test. Data are presented as mean values +/- 95% confidence intervals. The bar charts represent the mean value of AUCs and error bar represent the 95% confidence intervals. *: $P < 0.01$, **: $P < 0.001$. Source data are provided as a Source Data file.

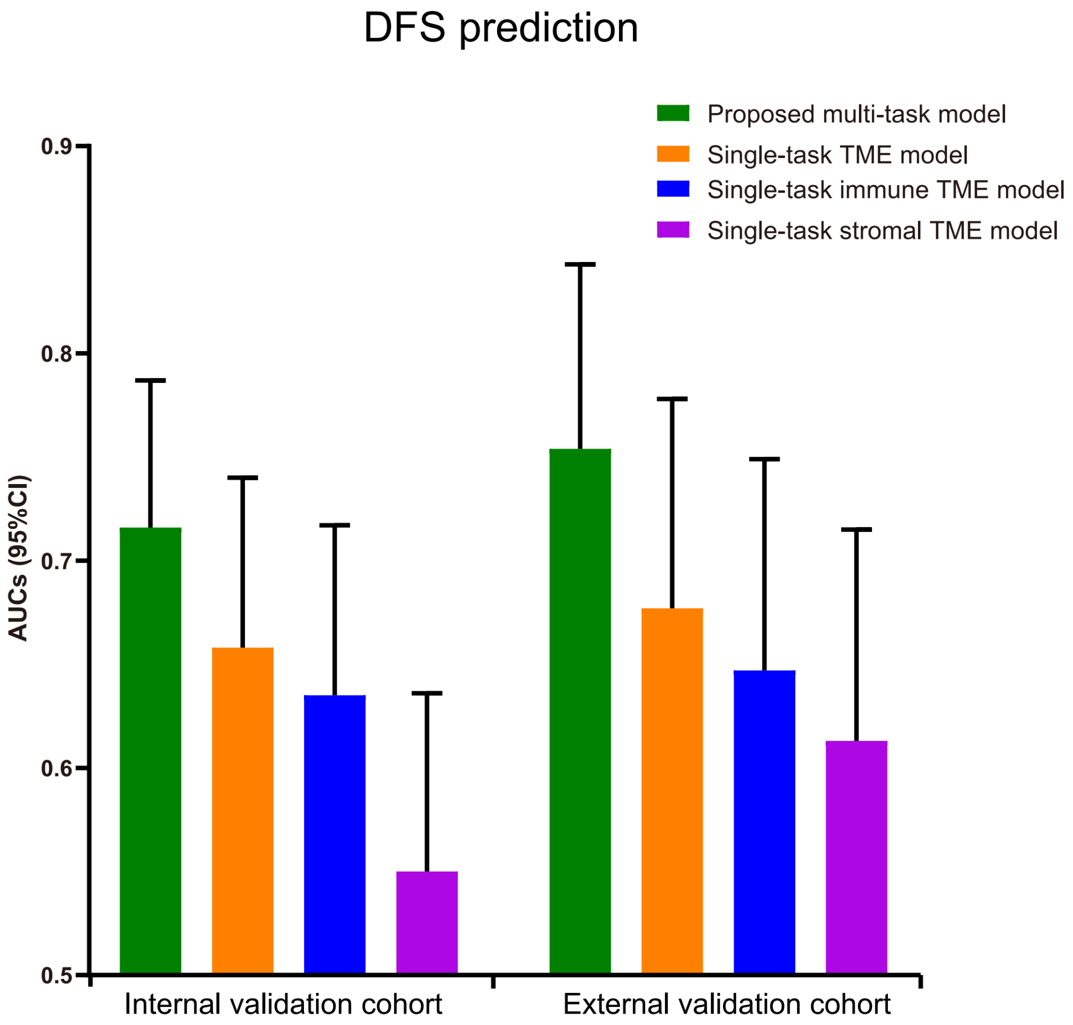

Supplementary Figure 16. Comparison of the proposed multi-task learning approach with the single-task learning for prediction of TME class, immune TME, and stromal TME.

AUC: area under the ROC curves, TME：tumor microenvironment, DFS: disease-free survival. Internal validation cohort (n=838), External validation cohort (n=1310). The bar charts represent the mean value of AUCs and error bar represent the 95% confidence intervals. Source data are provided as a Source Data file.

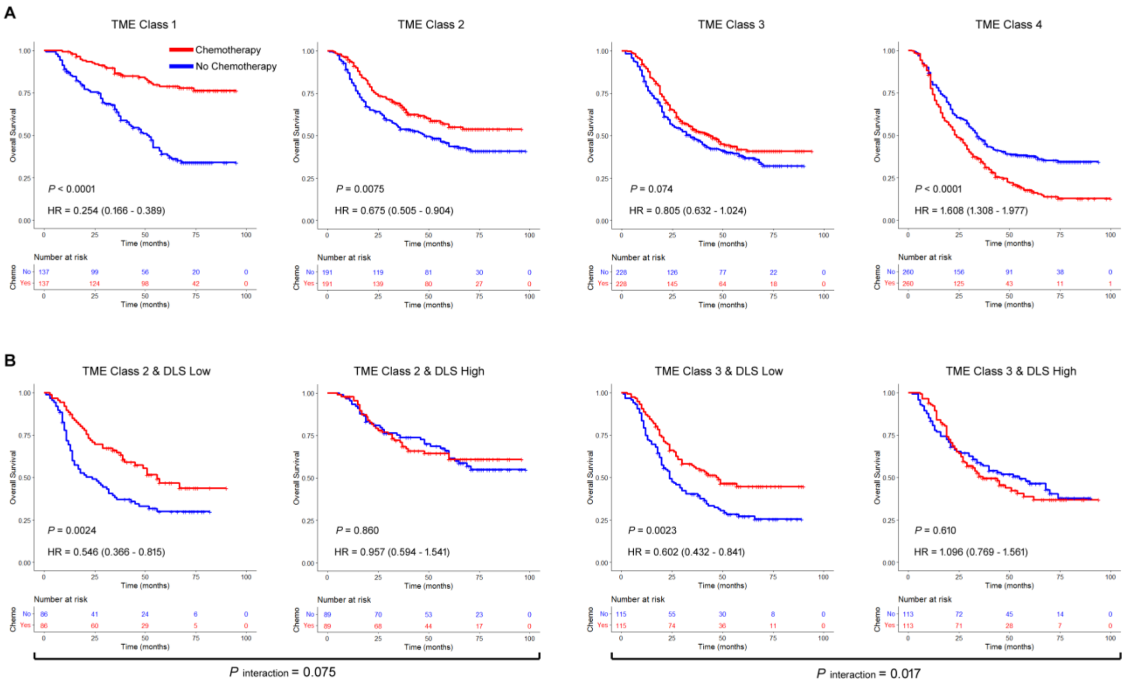

Supplementary Figure 17. Relationship between the predicted TME classes, DLS score and survival classes and benefit from adjuvant chemotherapy in patients with stage II and III gastric cancer. Kaplan-Meier survival curves of overall survival (OS) for patients with gastric cancer in different predicted TME classes, which were stratified by the receipt of chemotherapy. A, TME class 1 (n = 274), TME class 2 (n = 382), TME class 3 (n = 456), TME class 4 (n = 520). B, combined the predicted TME classes and DLS score. Chemo: chemotherapy. Comparisons of the survival curves were performed with a two-sided log-rank test. Source data are provided as a Source Data file.

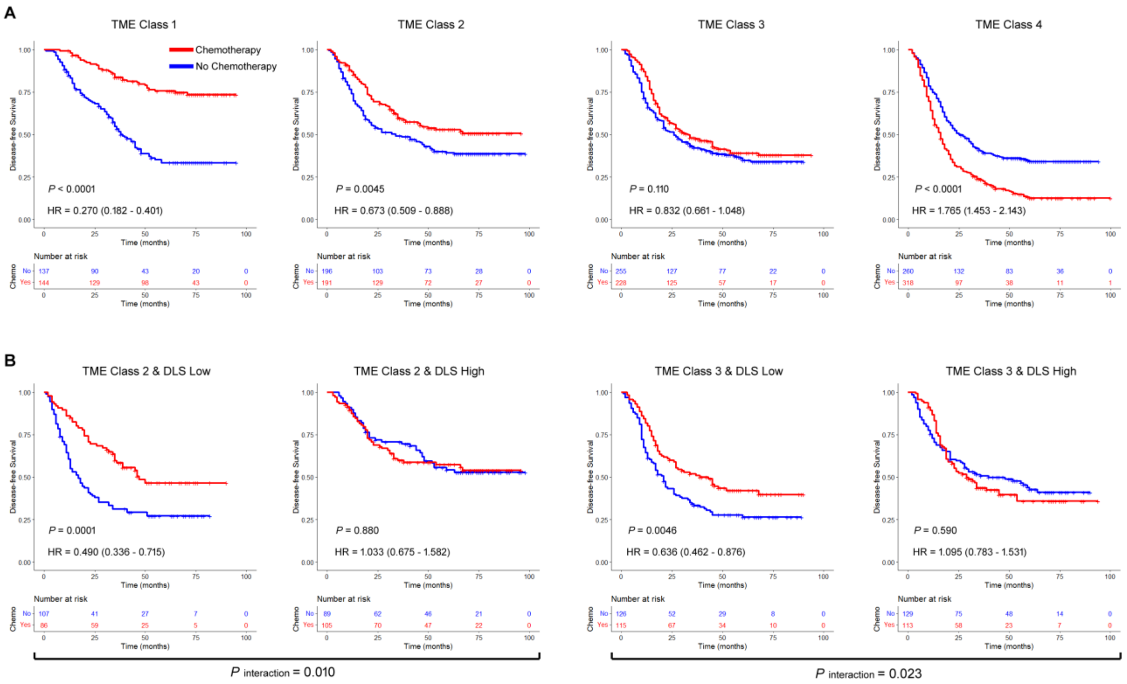

Supplementary Figure 18. Relationship between the predicted TME classes, DLS score and survival classes and benefit from adjuvant chemotherapy in unmatched patients with stage II and III gastric cancer.

Kaplan-Meier survival curves of disease-free survival (DFS) for patients with gastric cancer in different predicted TME classes, which were stratified by the receipt of chemotherapy. A, TME class 1 (n = 281), TME class 2 (n = 387), TME class 3 (n = 483), TME class 4 (n = 578). B, combined the predicted TME classes and DLS score. Chemo: chemotherapy. Comparisons of the survival curves were performed with a two-sided log-rank test. Source data are provided as a Source Data file.

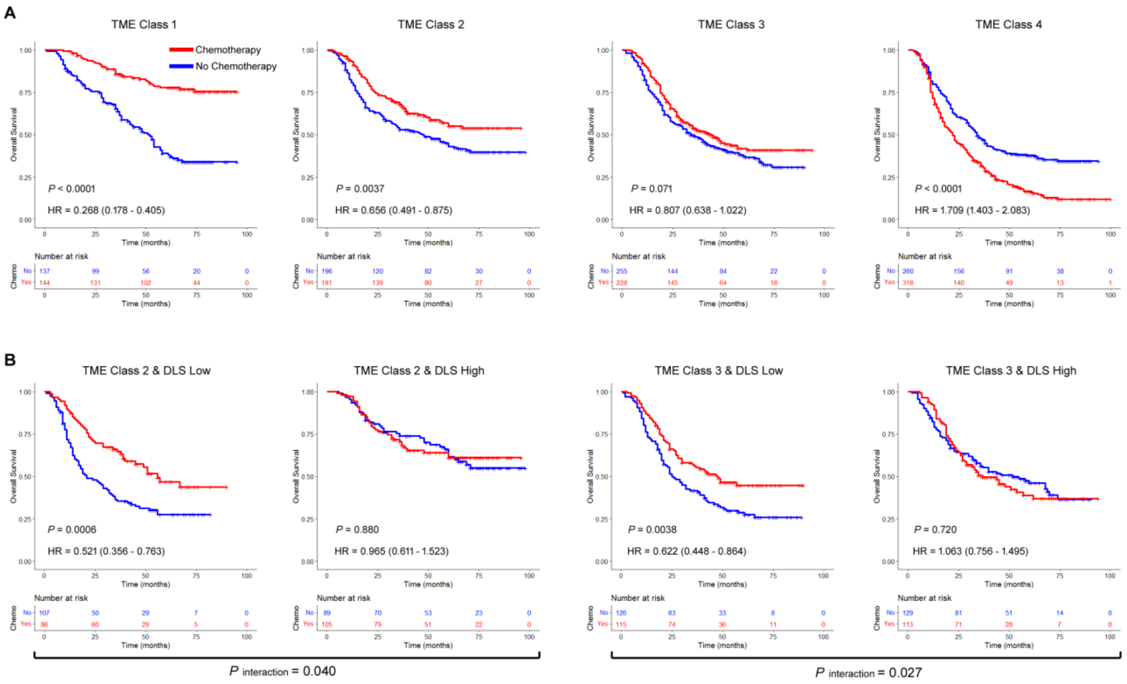

Supplementary Figure 19. Relationship between the predicted TME classes, DLS score and survival classes and benefit from adjuvant chemotherapy in unmatched patients with stage II and III gastric cancer.

Kaplan-Meier survival curves of overall survival (OS) for patients with gastric cancer in different predicted TME classes, which were stratified by the receipt of chemotherapy. A, TME class 1 (n = 281), TME class 2 (n = 387), TME class 3 (n = 483), TME class 4 (n = 578). B, combined the predicted TME classes and DLS score. Chemo: chemotherapy. Comparisons of the survival curves were performed with a two-sided log-rank test. Source data are provided as a Source Data file.

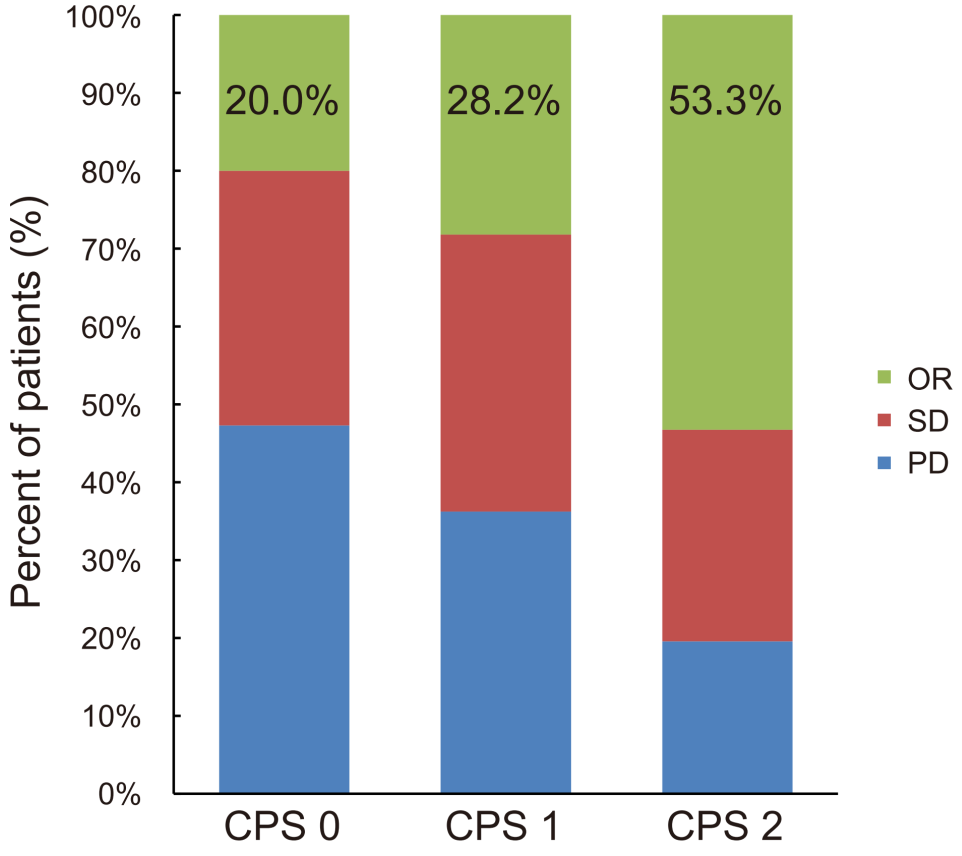

Supplementary Figure 20. Proportion of response to anti-PD-1 immunotherapy response in different CPS (n=296). Source data are provided as a Source Data file.

Multivariate logistic regression analysis for objective response in patients with different CPS.

Subgroup	Number of patients	OR (95% CI)	P value
**CPS High**			
**TME Class 1 vs. 3/4**	24/47	8.824 (2.586 − 30.108)	0.001
**TME Class 2 vs. 3/4**	21/47	2.353 (0.824 − 6.719)	0.110
**CPS Moderate**			
**TME Class 1 vs. 3/4**	25/97	27.321 (8.746 − 85.353)	<0.001
**TME Class 2 vs. 3/4**	27/97	25.714 (8.476 − 78.013)	<0.001
**CPS Low**			
**TME Class 1 vs. 3/4**	9/37	2.583 (0.502 − 13.298)	0.256
**TME Class 2 vs. 3/4**	9/37	1.476 (0.244 − 8.915)	0.671

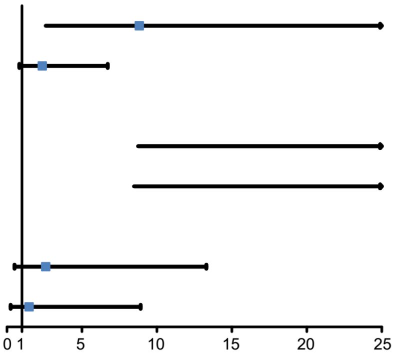

Supplementary Figure 21. Multivariate logistic regression analysis for objective response in patients with different CPS (n=296). Blue dot represents the HR value. Error bars represent the 95% confidence intervals. *P* values reported are two-tailed from logistic regression analyses. Source data are provided as a Source Data file.

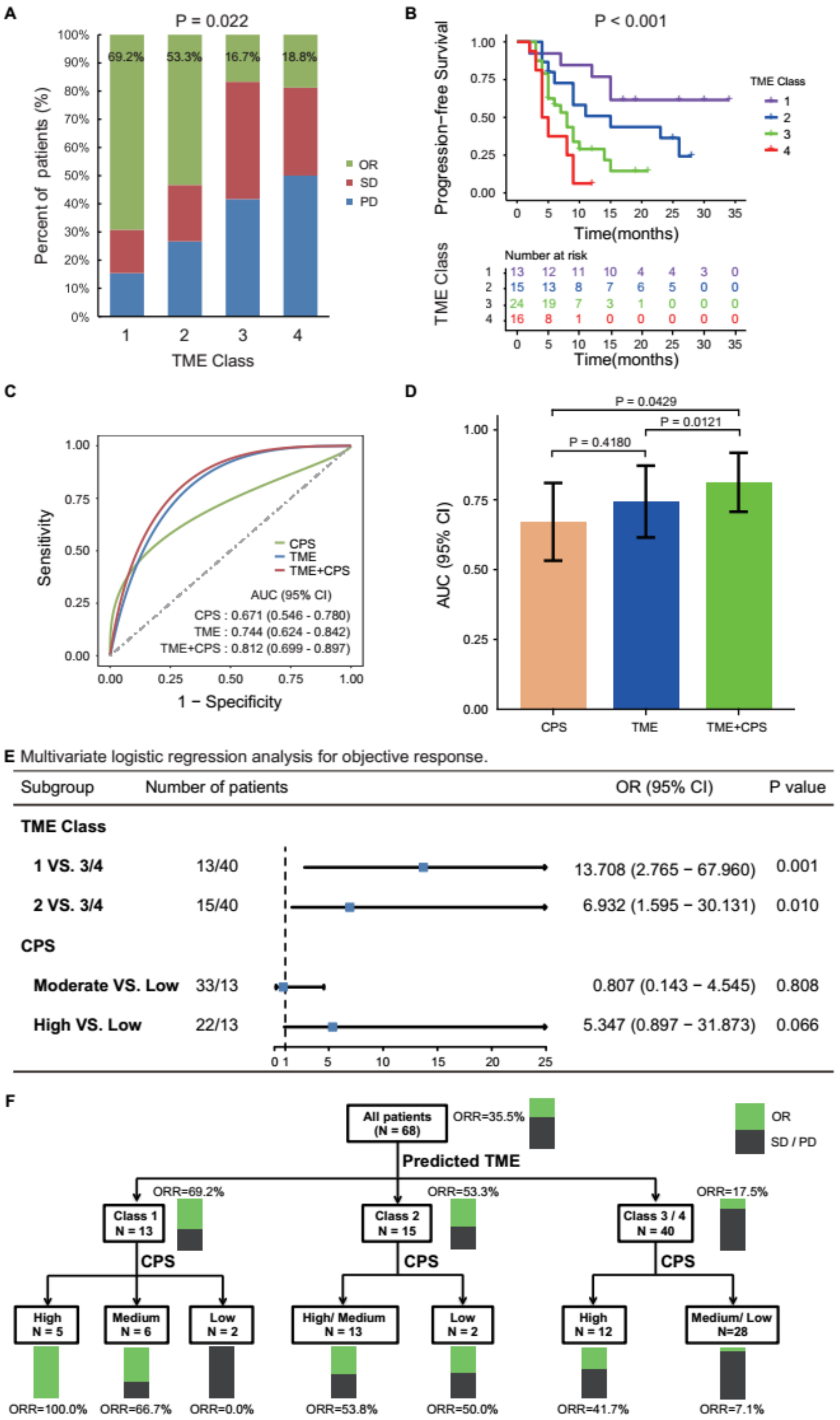

A
P = 0.022
Percent of patients (%)
69.2%
53.3%
16.7%
18.8%
OR
SD
PD
TME Class
B
P < 0.001
Progression−free Survival
TME Class
Time(months)
Number at risk
1 13 12 11 10 4 4 3 0
2 15 13 8 7 6 5 0 0
3 24 19 7 3 1 0 0 0
4 16 8 1 0 0 0 0 0
C
Sensitivity
1 − Specificity
CPS
TME
TME+CPS
AUC (95% CI)
CPS : 0.671 (0.546 - 0.780)
TME : 0.744 (0.624 - 0.842)
TME+CPS : 0.812 (0.699 - 0.897)
D
AUC (95% CI)
P = 0.0429
P = 0.0121
P = 0.4180
CPS
TME
TME+CPS
E Multivariate logistic regression analysis for objective response.
Subgroup
Number of patients
OR (95% CI)
P value
TME Class
1 VS. 3/4
13/40
13.708 (2.765 − 67.960)
0.001
2 VS. 3/4
15/40
6.932 (1.595 − 30.131)
0.010
CPS
Moderate VS. Low
33/13
0.807 (0.143 − 4.545)
0.808
High VS. Low
22/13
5.347 (0.897 − 31.873)
0.066
F
All patients (N = 68)
ORR=35.5%
OR
SD / PD
Predicted TME
ORR=69.2%
ORR=53.3%
ORR=17.5%
Class 1 N = 13
Class 2 N = 15
Class 3 / 4 N = 40
CPS
High N = 5
Medium N = 6
Low N = 2
High/ Medium N = 13
Low N = 2
High N = 12
Medium/ Low N=28
ORR=100.0%
ORR=66.7%
ORR=0.0%
ORR=53.8%
ORR=50.0%
ORR=41.7%
ORR=7.1%

Supplementary Figure 22. Performance of the deep learning model in predicting response and outcomes in patients treated with single-agent anti-PD-1 immunotherapy.

(A), Response rates in patients of four TME classes predicted by the deep learning model; (B), Progression-free survival in patients of four predicted TME classes; (C), Receiver operator characteristic (ROC) curves of the predicted TME classes, CPS and composite models combining TME classes and CPS for predicting immunotherapy response (n=68); (D), AUC values of the predicted TME classes, CPS and composite models combining TME classes and CPS for predicting immunotherapy response (n=68); (E), Forest plot for the multivariate logistic regression analysis for objective response; (F), Decision tree combining the predicted TME classes and CPS. AUC: area under the receiver operator characteristic curve. CPS: combined positive score of PDL1 expression. OR: objective response (complete and partial response); SD: stable disease; PD: progressive disease. Comparisons of the survival curves were performed with a two-sided log-rank test. Comparisons of the bar plot were performed with a two-sided t (unpaired) test. P values reported in (E) are two-tailed from logistic regression analyses. Blue dot represents the HR value. The bar charts represent the mean value of AUCs and error bar represent the 95% confidence intervals in (D). Error bars in (E) represent the 95% confidence intervals. Source data are provided as a Source Data file.

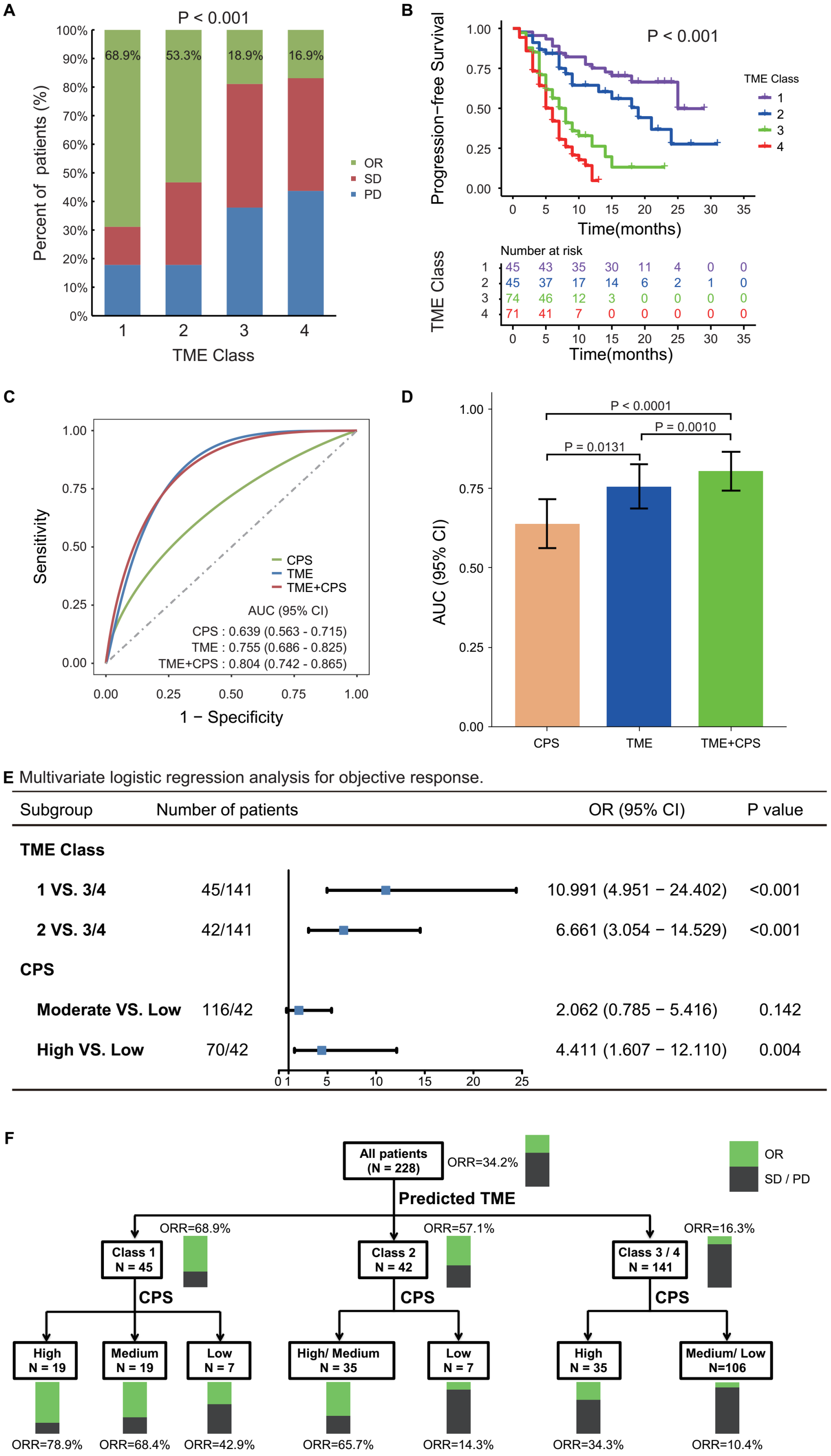

A
P < 0.001
Percent of patients (%)
100%
90%
80%
70%
60%
50%
40%
30%
20%
10%
0%
68.9%
53.3%
18.9%
16.9%
OR
SD
PD
1
2
3
4
TME Class
B
Progression−free Survival
1.00
0.75
0.50
0.25
0.00
P < 0.001
TME Class
1
2
3
4
0 5 10 15 20 25 30 35
Time(months)
Number at risk
TME Class
1 45 43 35 30 11 4 0 0
2 45 37 17 14 6 2 1 0
3 74 46 12 3 0 0 0 0
4 71 41 7 0 0 0 0 0
0 5 10 15 20 25 30 35
Time(months)
C
Sensitivity
1.00
0.75
0.50
0.25
0.00
CPS
TME
TME+CPS
AUC (95% CI)
CPS : 0.639 (0.563 - 0.715)
TME : 0.755 (0.686 - 0.825)
TME+CPS : 0.804 (0.742 - 0.865)
0.00 0.25 0.50 0.75 1.00
1 − Specificity
D
AUC (95% CI)
1.00
0.75
0.50
0.25
0.00
P < 0.0001
P = 0.0010
P = 0.0131
CPS
TME
TME+CPS
E Multivariate logistic regression analysis for objective response.
Subgroup
Number of patients
OR (95% CI)
P value
TME Class
1 VS. 3/4
45/141
10.991 (4.951 − 24.402)
<0.001
2 VS. 3/4
42/141
6.661 (3.054 − 14.529)
<0.001
CPS
Moderate VS. Low
116/42
2.062 (0.785 − 5.416)
0.142
High VS. Low
70/42
4.411 (1.607 − 12.110)
0.004
0 1 5 10 15 20 25
F
All patients
(N = 228)
ORR=34.2%
OR
SD / PD
Predicted TME
ORR=68.9%
Class 1
N = 45
ORR=57.1%
Class 2
N = 42
ORR=16.3%
Class 3 / 4
N = 141
CPS
High
N = 19
Medium
N = 19
Low
N = 7
High/ Medium
N = 35
Low
N = 7
High
N = 35
Medium/ Low
N=106
ORR=78.9%
ORR=68.4%
ORR=42.9%
ORR=65.7%
ORR=14.3%
ORR=34.3%
ORR=10.4%

Supplementary Figure 23. Performance of the deep learning model in predicting response and outcomes in patients treated with combination therapy of anti-PD-1 immunotherapy and chemotherapy.

(A), Response rates in patients of four TME classes predicted by the deep learning model; (B), Progression-free survival in patients of four predicted TME classes; (C), Receiver operator characteristic (ROC) curves of the predicted TME classes, CPS and composite models combining TME classes and CPS for predicting immunotherapy response (n=228); (D), AUC values of the predicted TME classes, CPS and composite models combining TME classes and CPS for predicting immunotherapy response (n=228); (E), Forest plot for the multivariate logistic regression analysis for objective response; (F), Decision tree combining the predicted TME classes and CPS. AUC: area under the receiver operator characteristic curve. CPS: combined positive score of PDL1 expression. OR: objective response (complete and partial response); SD: stable disease; PD: progressive disease. Comparisons of the survival curves were performed with a two-sided log-rank test. Comparisons of the bar plot were performed with a two-sided t (unpaired) test. P values reported in (E) are two-tailed from logistic regression analyses. Blue dot represents the HR value. The bar charts represent the mean value of AUCs and error bar represent the 95% confidence intervals in (D). Error bars in (E) represent the 95% confidence intervals. Source data are provided as a Source Data file.

Supplementary Tables

Supplementary Table 1. Antibody sources and staining conditions.

Markers	Main target	Antibody source	Species	Dilution	DAB dyeing time	Antigen Retrieval	Cellular localization
CD3	Pan T lymphocyte	NeoMarker, clone SP7	Rabbit monoclonal	1:300	1.0 min	Citrate buffer (pH 6.0) microwave 20min	Membranous
CD8	Cytotoxic T lymphocyte	NeoMarker, clone SP16	Rabbit monoclonal	1:200	1.5 min	Citrate buffer (pH 6.0) microwave 20min	Membranous
CD45RO	Memory T lymphocyte	Invitrogen, clone UCHL1	Mouse monoclonal	1:400	1.0 min	Citrate buffer (pH 6.0) microwave 20min	Membranous
CD66b	Neutrophil	BD Pharmingen	Mouse monoclonal	1:200	1.0 min	Citrate buffer (pH 6.0) microwave 20min	Membranous
POSTN	Fibroblast	Abcam,ab92460	Rabbit monoclonal	1:200	30 secs	Citrate buffer (pH 6.0) microwave 20min	Cytoplasmic

min: minute; sec: second. DAB: diaminobenzidine.

Supplementary Table 2. Performance of the deep learning signature for the assessment of tumor stroma classes in the training and validation cohorts

Predicted TME class	Accuracy (95% CI)	Sensitivity (95% CI)	Specificity (95% CI)	PPV(95% CI)	NPV(95% CI)
**Training SMU-1 cohort**					
**1**	0.891 (0.858-0.924)	69.89 (59.5 - 79.0)	96.08 (92.9 - 98.1)	86.7 (76.8 - 93.5)	89.7 (85.5 - 93.1)
**2**	0.931 (0.904-0.958)	69.89 (59.5 - 79.0)	96.08 (92.9 - 98.1)	86.7 (76.8 - 93.5)	89.7 (85.5 - 93.1)
**3**	0.911 (0.881-0.941)	75.00 (63.7 - 84.2)	95.59 (92.4 - 97.7)	82.6 (71.5 - 90.7)	93.2 (89.6 - 95.9)
**4**	0.888 (0.855-0.921)	92.47 (85.1 - 96.9)	87.45 (82.7 - 91.3)	72.9 (63.9 - 80.7)	97.0 (93.8 - 98.8)
**Internal validation SMU-2 cohort**					
**1**	0.901 (0.860-0.942)	70.59 (56.2 - 82.5)	96.69 (92.4 - 98.9)	87.8 (73.6 - 96.0)	90.7 (85.1 - 94.7)
**2**	0.926 (0.890-0.962)	82.50 (67.2 - 92.7)	95.06 (90.5 - 97.8)	80.5 (65.1 - 91.2)	95.7 (91.2 - 98.2)
**3**	0.921 (0.884-0.958)	86.00 (73.3 - 94.2)	94.08 (89.1 - 97.3)	82.7 (69.7 - 91.8)	95.3 (90.6 - 98.1)
**4**	0.916 (0.878-0.954)	91.80 (81.9 - 97.3)	91.49 (85.6 - 95.5)	82.4 (71.2 - 90.5)	96.3 (91.5 - 98.8)
**External validation SYSUCC-1 cohort**					
**1**	0.920 (0.872-0.968)	75.00 (55.1 - 89.3)	96.91 (91.2 - 99.4)	87.5 (67.6 - 97.3)	93.1 (86.2 - 97.2)
**2**	0.944 (0.904-0.984)	84.62 (65.1 - 95.6)	96.97 (91.4 - 99.4)	88.0 (68.3 - 97.6)	96.0 (90.1 - 98.9)
**3**	0.904 (0.852-0.956)	86.11 (70.5 - 95.3)	96.13 (84.5 - 96.8)	81.6 (65.4 - 92.4)	94.3 (87.0 - 98.1)
**4**	0.912 (0.862-0.962)	88.57 (73.3 - 96.8)	92.22 (84.6 - 96.8)	81.6 (65.4 - 92.4)	95.4 (88.6 - 98.7)

All numbers are shown for each class and calculated from one-vs-rest comparison.

Supplementary Table 3. Univariate association of DLS, clinicopathological characteristics with disease-free and overall survival in the training SMU-1 cohort.

**Variables**	**Disease-free survival**		**Overall survival**	
	HR (95%CI)	*p*	HR (95%CI)	*p*
**DLS**	**0.121 (0.063-0.233)**	**<0.0001**	**0.116 (0.061-0.222)**	**<0.0001**
Age(years) (≥60 vs. <60)	1.192(0.912-1.559)	0.199	1.138 (0.086-1.504)	0.364
Gender (male vs. female)	1.275 (0.957-1.700)	0.097	1.320 (0.978-1.782)	0.069
Tumor size (>4 cm vs. ≤4 cm)	1.567 (1.202-2.043)	0.001	1.450 (1.101-1.909)	0.008
Tumor location	0.917 (0.788-1.066)	0.259	0.940 (0.802-1.101)	0.441
Differentiation	1.342 (1.099-1.638)	0.004	1.367 (1.108-1.686)	0.004
Lauren type	1.220 (0.934-1.593)	0.144	1.205(0.913-1.589)	0.187
CEA(ng/ml)	1.620 (1.089-2.410)	0.017	1.580 (1.046-2.385)	0.03
CA199(U/ml)	1.530 (1.070-2.187)	0.020	1.358 (0.931-1.981)	0.112
Depth of invasion	1.313 (1.212-1.422)	<0.0001	1.312 (1.207-1.426)	<0.0001
Lymph node metastasis	1.381 (1.266-1.506)	<0.0001	1.404 (1.284-1.536)	<0.0001
Distant metastasis	3.089(1.877-5.082)	<0.0001	2.231 (1.339-3.719)	0.002
Chemotherapy	0.897 (0.689-1.169)	0.423	0.834 (0.634-1.098)	0.196

HR: hazard ratio, *P* values reported are two-tailed from Cox proportional hazard regression analyses.

Supplementary Table 4. Univariate association of DLS, clinicopathological characteristics with disease-free and overall survival in the internal validation SMU-2 cohort.

Variables	Disease-free survival		Overall survival	
	HR (95%CI)	*p*	HR (95%CI)	*p*
**DLS**	**0.080 (0.034-0.187)**	**<0.0001**	**0.056 (0.023-0.132)**	**<0.0001**
Age(years) (≥60 vs. <60)	1.020(0.758-1.372)	0.896	1.029 (0.762-1.390)	0.852
Gender (male vs. female)	1.081 (0.743-1.573)	0.685	1.098(0.754-1.599)	0.626
Tumor size (>4 cm vs. ≤4 cm)	1.973 (1.384-2.812)	<0.0001	1.930 (1.351-2.757)	<0.0001
Tumor location	1.110 (0.913-1.349)	0.296	1.094 (0.900-1.329)	0.368
Differentiation	1.406 (1.034-1.912)	0.030	1.469 (1.078-2.002)	0.015
Lauren type	1.368 (0.975-1.918)	0.069	1.419 (1.011-1.993)	0.043
CEA(ng/ml)	1.746 (1.083-2.815)	0.022	1.626 (0.999-2.647)	0.051
CA199(U/ml)	1.483 (0.939-2.343)	0.091	1.301 (0.817-2.075)	0.268
Depth of invasion	1.279 (1.145-1.429)	<0.0001	1.264 (1.132-1.411)	<0.0001
Lymph node metastasis	1.519 (1.348-1.711)	<0.0001	1.502 (1.334-1.692)	<0.0001
Distant metastasis	3.731 (1.728-8.060)	0.001	3.978 (1.844-8.580)	<0.0001
Chemotherapy	0.888 (0.631-1.250)	0.497	0.872 (0.619-1.229)	0.435

HR: hazard ratio, *P* values reported are two-tailed from Cox proportional hazard regression analyses.

Supplementary Table 5. Univariate association of DLS, clinicopathological characteristics with disease-free and overall survival in the internal validation SMU-3 cohort.

Variables	Disease-free survival		Overall survival	
	HR (95%CI)	*p*	HR (95%CI)	*p*
**DLS**	**0.261 (0.171-0.399)**	**<0.0001**	**0.228 (0.146-0.356)**	**<0.0001**
Age(years) (≥60 vs. <60)	1.131 (1.026-1.247)	0.013	1.217 (1.099-1.348)	<0.0001
Gender (male vs. female)	1.054 (0.848-1.311)	0.635	1.139 (0.904-1.435)	0.27
Tumor size (>4 cm vs. ≤4 cm)	1.155 (0.948-1.406)	0.152	1.118(0.908-1.376)	0.292
Tumor location	0.981 (0.875-1.099)	0.738	0.987(0.874-1.113)	0.827
Differentiation	1.269 (1.117-1.442)	<0.0001	1.304 (1.138-1.494)	<0.0001
Lauren type	1.095 (0.897-1.336)	0.373	1.130 (0.916-1.395)	0.253
CEA(ng/ml)	1.699 (1.302-2.218)	<0.0001	1.831 (1.392-2.410)	<0.0001
CA199(U/ml)	1.645 (1.301-2.079)	<0.0001	1.647 (1.289-2.103)	<0.0001
Depth of invasion	1.378 (1.289-1.474)	<0.0001	1.424 (1.323-1.533)	<0.0001
Lymph node metastasis	1.169 (1.094-1.249)	<0.0001	1.148 (1.070-1.231)	<0.0001
Distant metastasis	2.812 (2.086-3.790)	<0.0001	2.660 (1.949-3.629)	<0.0001
Chemotherapy	0.887 (0.727-1.083)	0.238	0.853 (0.692-1.051)	0.136

HR: hazard ratio, *P* values reported are two-tailed from Cox proportional hazard regression analyses.

Supplementary Table 6. Univariate association of DLS, clinicopathological characteristics with disease-free and overall survival in the external validation SYSUCC-1 cohort.

Variables	Disease-free survival		Overall survival	
	HR (95%CI)	*p*	HR (95%CI)	*p*
**DLS**	**0.042 (0.014-0.127)**	**<0.0001**	**0.030 (0.010-0.097)**	**<0.0001**
Age(years) (≥60 vs. <60)	1.348 (0.841-2.161)	0.215	1.413 (0.879-2.271)	0.154
Gender (male vs. female)	1.040 (0.609-1.779)	0.885	0.970 (0.567-1.661)	0.912
Tumor size (>4 cm vs. ≤4 cm)	2.558 (1.507-4.342)	0.001	1.808 (1.637-4.818)	<0.0001
Tumor location	0.822 (0.643-1.052)	0.119	0.784 (0.610-1.009)	0.059
Differentiation	0.977(0.573-1.666)	0.932	0.941 (0.554-1.600)	0.823
Lauren type	0.892 (0.552-1.440)	0.639	0.809 (0.500-1.309)	0.388
CEA(ng/ml)	1.178 (0.656-2.118)	0.583	1.238 (0.688-2.227)	0.476
CA199(U/ml)	2.119 (1.156-3.884)	0.015	2.459 (1.338-4.521)	0.004
Depth of invasion	1.199 (1.040-1.383)	0.013	1.236 (1.068-1.430)	0.004
Lymph node metastasis	1.251 (1.064-1.471)	0.007	1.282 (1.086-1.513)	0.003
Distant metastasis	2.071 (0.990-4.332)	0.053	2.459 (1.170-5.170)	0.018
Chemotherapy	1.322 (0.827-2.112)	0.244	1.332 (0.831-2.137)	0.234

HR: hazard ratio, *P* values reported are two-tailed from Cox proportional hazard regression analyses.

Supplementary Table 7. Univariate association of DLS, clinicopathological characteristics with disease-free and overall survival in the external validation SYSUCC-2 cohort.

Variables	Disease-free survival		Overall survival	
	HR (95%CI)	*p*	HR (95%CI)	*p*
**DLS**	**0.130 (0.089-0.189)**	**<0.0001**	**0.144 (0.099-0.208)**	**<0.0001**
Age(years) (≥60 vs. <60)	1.305 (1.100-1.548)	0.002	1.302 (1.096-1.546)	0.003
Gender (male vs. female)	1.036 (0.862-1.245)	0.708	1.056 (0.877-1.271)	0.566
Tumor size (>4 cm vs. ≤4 cm)	1.903 (1.575-2.299)	<0.0001	1.896 (1.567-2.293)	<0.0001
Tumor location	0.924 (0.843-1.012)	0.09	0.924(0.843-1.014)	0.094
Differentiation	1.300 (1.050-1.609)	0.016	1.308 (1.055-1.621)	0.014
Lauren type	1.329 (0.101-1.605)	0.003	1.346 (1.113-1.627)	0.002
CEA(ng/ml)	1.599 (1.315-1.945)	<0.0001	1.587 (1.303-1.933)	<0.0001
CA199(U/ml)	1.818 (1.520-2.200)	<0.0001	1.847(1.525-2.237)	<0.0001
Depth of invasion	1.427(1.339-1.521)	<0.0001	1.417 (1.330-1.511)	<0.0001
Lymph node metastasis	1.492 (1.403-1.587)	<0.0001	1.487(1.398-1.581)	<0.0001
Distant metastasis	3.542 (2.814-4.458)	<0.0001	3.581 (2.843-4.510)	<0.0001
Chemotherapy	1.011 (0.852-1.200)	0.897	1.044 (0.879-1.241)	0.622

HR: hazard ratio, *P* values reported are two-tailed from Cox proportional hazard regression analyses.

Supplementary Table 8. Univariate association of DLS, clinicopathological characteristics with disease-free and overall survival in external validation Stanford cohort.

Variables	Disease-free survival		Overall survival	
	HR (95%CI)	*p*	HR (95%CI)	*p*
**DLS**	**0.430 (0.222-0.835)**	**0.013**	**0.450 (0.216-0.939)**	**0.033**
Age(years) (≥60 vs. <60)	1.695(0.872-3.294)	0.12	1.705 (0.815-3.568)	0.157
Gender (male vs. female)	1.326 (0.774-2.270)	0.304	1.101(0.606-2.000)	0.751
Tumor size (>4 cm vs. ≤4 cm)	2.560 (1.452-4.514)	0.001	2.108 (1.141-3.897)	0.017
Tumor location	0.844 (0.611-1.166)	0.304	0.891 (0.622-1.276)	0.530
Differentiation	1.391 (0.825-2.347)	0.216	1.238(0.715-2.144)	0.446
Lauren type	1.113(0.833-1.486)	0.469	1.123(0.814-1.550)	0.480
Depth of invasion	1.462 (1.185-1.804)	<0.0001	1.355 (1.074-1.709)	0.010
Lymph node metastasis	1.517 (1.273-1.809)	<0.0001	1.450 (1.191-1.766)	<0.0001
Distant metastasis	16.875(6.089-46.772)	<0.0001	13.430 (4.874-37.007)	<0.0001
Chemotherapy	1.173 (0.716-1.922)	0.525	0.764 (0.431-1.353)	0.356

HR: hazard ratio, *P* values reported are two-tailed from Cox proportional hazard regression analyses.

Supplementary Table 9. The integrated Brier score of each model in the validation cohorts

Variable	Internal validation cohorts		External validation cohorts	
	DFS	OS	DFS	OS
Integrated nomogram*	0.179	0.152	0.168	0.149
Clinical model	0.184	0.163	0.175	0.159
Stage	0.185	0.165	0.174	0.158
Image based survival score	0.198	0.175	0.184	0.166

IBS: integrated Brier score. Integrated nomogram*: combined the image-based survival score and clinical pathological variables. Lower values of IBS indicated better model performance

Supplementary Table 10. Net reclassification improvement by comparing the integrated nomogram with the image based survival score and clinical model.

Patients	Disease-free survival		Overall survival	
	NRI (95% CI)	*p*	NRI (95% CI)	*p*
**Integrated nomogram* *vs.***				
**Image based survival score**				
SMU validation cohorts	0.345 (0.219-0.409)	<0.001	0.324 (0.183-0.416)	<0.001
SYSUCC validation cohorts	0.353 (0.267-0.408)	<0.001	0.336 (0.261-0.403)	<0.001
**Integrated nomogram *vs.* Clinical model**				
SMU validation cohorts	0.156 (0.005-0.286)	0.030	0.246 (0.105-0.349)	0.010
SYSUCC validation cohorts	0.187 (0.064-0.272)	<0.001	0.187 (0.068-0.277)	<0.001

Integrated nomogram: combined the image-based survival score and clinical pathological variables. NRI: Net reclassification improvement.

Supplementary Table 11. Multivariate analysis for association with chemotherapy response.

**Variables**	OR (95% CI)	*P*
**TME class**		<0.0001
1	Reference	
2	0.257 (0.138-0.480)	<0.0001
3	0.170 (0.093-0.312)	<0.0001
4	0.054 (0.028-0.105)	<0.0001
**Depth of invasion**		.027
pT1	Reference	
pT2	0.604 (0.341-1.068)	.083
pT3	0.724 (0.416-1.257)	.251
pT4a	0.627 (0.371-1.060)	.082
pT4b	0.393 (0.222-0.693)	.001
**Lymph node metastasis**		.050
pN0	Reference	
pN1	0.620 (0.158-2.427)	.492
pN2	0.354 (0.103-1.220)	.100
pN3a	0.396 (0.117-1.335)	.135
pN3b	0.239 (0.068-0.841)	.026

P values are two-tailed from logistic test.
