# Supplementary material for: Biology-guided deep learning predicts prognosis and cancer immunotherapy response
Source: Nat Commun. 2023 Aug 23;14:5135. doi: 10.1038/s41467-023-40890-x (PMC10447467; doi:10.1038/s41467-023-40890-x)
Supplement: Supplementary file 3 — Description of Additional Supplementary Files [file 41467_2023_40890_MOESM3_ESM.pdf]

Description of Additional Supplementary Files

File Name: Supplementary Data 1
Description: Characteristics of patients in the training, internal and external validation cohorts

File Name: Supplementary Data 2
Description: Characteristics of patients in the anti-PD1 immunotherapy cohort.

File Name: Supplementary Data 3
Description: Clinical characteristics of patients according to the predicted TME classes in the training SMU-1 cohort.

File Name: Supplementary Data 4
Description: Clinical characteristics of patients according to the predicted TME classes in the internal validation SMU-2 cohort.

File Name: Supplementary Data 5
Description: Clinical characteristics of patients according to the predicted TME classes in the internal validation SMU-3 cohort.

File Name: Supplementary Data 6
Description: Clinical characteristics of patients according to the predicted TME classes in the external validation SYSUCC-1 cohort.

File Name: Supplementary Data 7
Description: Clinical characteristics of patients according to the predicted TME classes in the external validation SYSUCC-2 cohort.

File Name: Supplementary Data 8
Description: Clinical characteristics of patients according to the predicted survival score in the training and internal validation cohorts.

File Name: Supplementary Data 9
Description: Clinical characteristics of patients according to the DLS in the training, internal and external validation cohorts.

File Name: Supplementary Data 10
Description: Clinical characteristics of patients according to the DLS in the training, internal and external validation Stanford cohort.

File Name: Supplementary Data 11
Description: Multivariate Cox Regression analyses for disease-free survival and overall survival in patients with gastric cancer.

File Name: Supplementary Data 12
Description: Clinical characteristics of patients according to the chemotherapy in stage II and III patients after PSM.

File Name: Supplementary Data 13
Description: Clinical characteristics of patients according to the chemotherapy in stage II and III patients before PSM.
