# Supplementary material for: Biology-guided deep learning predicts prognosis and cancer immunotherapy response
Source: Nat Commun. 2023 Aug 23;14:5135. doi: 10.1038/s41467-023-40890-x (PMC10447467; doi:10.1038/s41467-023-40890-x)
Supplement: Supplementary file 17 — Reporting Summary [file 41467_2023_40890_MOESM17_ESM.pdf]

nature portfolio

Corresponding author(s): Ruijiang Li

Last updated by author(s): Aug 14, 2023

Reporting Summary

Nature Portfolio wishes to improve the reproducibility of the work that we publish. This form provides structure for consistency and transparency in reporting. For further information on Nature Portfolio policies, see our Editorial Policies and the Editorial Policy Checklist.

Statistics

For all statistical analyses, confirm that the following items are present in the figure legend, table legend, main text, or Methods section.

n/a	Confirmed	
☐	☒	The exact sample size (*n*) for each experimental group/condition, given as a discrete number and unit of measurement
☐	☒	A statement on whether measurements were taken from distinct samples or whether the same sample was measured repeatedly
☐	☒	The statistical test(s) used AND whether they are one- or two-sided<br>*Only common tests should be described solely by name; describe more complex techniques in the Methods section.*
☐	☒	A description of all covariates tested
☐	☒	A description of any assumptions or corrections, such as tests of normality and adjustment for multiple comparisons
☐	☒	A full description of the statistical parameters including central tendency (e.g. means) or other basic estimates (e.g. regression coefficient) AND variation (e.g. standard deviation) or associated estimates of uncertainty (e.g. confidence intervals)
☐	☒	For null hypothesis testing, the test statistic (e.g. *F*, *t*, *r*) with confidence intervals, effect sizes, degrees of freedom and *P* value noted<br>*Give P values as exact values whenever suitable.*
☒	☐	For Bayesian analysis, information on the choice of priors and Markov chain Monte Carlo settings
☒	☐	For hierarchical and complex designs, identification of the appropriate level for tests and full reporting of outcomes
☒	☐	Estimates of effect sizes (e.g. Cohen's *d*, Pearson's *r*), indicating how they were calculated

Our web collection on statistics for biologists contains articles on many of the points above.

Software and code

Policy information about availability of computer code

Data collection	CT images were retrieved from the picture archiving and communication system {Carestream, Canada). Clinical data were collected from the electronic health record. Data were collected manually. No software was used.
Data analysis	The deep learning model was implemented using standard libraries in the open-source platform TensorFlow (version 1.14). SPSS version 21.0 (IBM) and R version 4.1.0 (http://www.r-project.org) were used for all statistical analyses. The "survivalROC" package Version: 1.0.3.1 was used to perform the time-dependent ROC analysis. Nomograms and calibration plots were generated using the "rms" package Version: 6.7-0 in R. Net reclassification improvement was computed using the "survIDINRI" package Version: 1.1-2 in R. The prediction error curves were obtained using the "pec" package Version: 2022.03.06 in R ("Boot- 632plus" split method with 1000 iterations). Classification and Regression Tree algorithm was implemented using the "caret" package (version 6.0-93) in R.<br>Custom source codes of model are available at: https://github.com/zzc623/ClassGastric.

For manuscripts utilizing custom algorithms or software that are central to the research but not yet described in published literature, software must be made available to editors and reviewers. We strongly encourage code deposition in a community repository (e.g. GitHub). See the Nature Portfolio guidelines for submitting code & software for further information.

Data

Policy information about availability of data

All manuscripts must include a data availability statement. This statement should provide the following information, where applicable:

- Accession codes, unique identifiers, or web links for publicly available datasets
- A description of any restrictions on data availability
- For clinical datasets or third party data, please ensure that the statement adheres to our policy

Source data are provided with this paper. The source data underlying Figures 3-8, supplementary Figures 3-23, and supplementary table 1, 2, 4-11, 13, 22 and 23 are provided as a Source Data file. The remaining data are available within the Article, Supplementary Information, or Source Data file. The de-identified individual patient data including CT images, tumor segmentations, IHC evaluation, clinicopathologic and follow-up data are available. A data transfer agreement is required that includes a brief research plan submitted by the user and data usage is restricted to non-commercial academic research purposes. Request for data access can be submitted to R.L. and will receive a response typically within 10 days. Data will be shared through cloud storage and available for 1 year once access has been granted.

Human research participants

Policy information about studies involving human research participants and Sex and Gender in Research.

Reporting on sex and gender	Gender was considered in the study design. The number of patients for both genders (male and female) is reported in Table 1 and Supplementary Table 1. Gender-specific analyses were performed, and results are shown in Supplementary Figures 6-7 Findings are applicable to both genders
Population characteristics	Detailed information about population characteristics is reported for each of the study cohorts in Supplementary Tables 1 and 2.
Recruitment	The training cohort and two internal validation cohorts included 348, 202, and 636 patients who were consecutively treated at Nanfang Hospital of Southern Medical University (Guangzhou, China) from January 1, 2005 to December 31, 2008, from January 1, 2009 to June 30, 2012, and from July 1, 2012 to December 31, 2016 respectively. The two external validation cohorts included 125 and 1062 patients consecutively treated at Sun Yat-sen University Cancer Center (SYSUCC) between June 1, 2007 and June 30, 2013. Another international external validation cohort included 123 patients treated at Stanford University Medical Center between August 1, 2000 and May 31, 2013. Additionally, we enrolled 303 advanced GC patients treated with anti-PD1 immunotherapy at Nanfang Hospital and Guangdong Provincial Hospital of Chinese Medicine between January 1, 2019 and July 31, 2021.
Ethics oversight	This study was approved by the Institutional Review Board of Nanfang Hospital of Southern Medical University (Guangzhou, China), Sun Yat-sen University Cancer Center (Guangzhou, China), Guangdong Provincial Hospital of Chinese Medicine (Guangzhou, China), and Stanford University School of Medicine (Stanford, CA, USA) .

Note that full information on the approval of the study protocol must also be provided in the manuscript.

Field-specific reporting

Please select the one below that is the best fit for your research. If you are not sure, read the appropriate sections before making your selection.

☒ Life sciences ☐ Behavioural & social sciences ☐ Ecological, evolutionary & environmental sciences

For a reference copy of the document with all sections, see nature.com/documents/nr-reporting-summary-flat.pdf

Life sciences study design

All studies must disclose on these points even when the disclosure is negative.

Sample size	A formal sample-size calculation was not performed in this study. Instead, sample size was determined empirically by collecting the largest number of samples possible given the pre-specified inclusion/exclusion criteria described in the Methods section. The training cohort and two internal validation cohorts included 348, 202, and 636 patients who were consecutively treated at Nanfang Hospital of Southern Medical University (Guangzhou, China) between 2005 and 2016. Other cohorts included patients who were consecutively treated at other participating centers and who met the inclusion criteria (detailed in patient recruitment).
Data exclusions	The data exclusion criteria are described in the Methods and Supplementary Information. In general, patients with missing or incomplete data or poor image quality were excluded.
Replication	We assessed the reproducibility of the model by independently testing its performance in two internal validation and three external validation cohorts.

Randomization	Not applicable as there was no experimental group in this study
Blinding	The clinical outcome data were available to the the investigator during model development, but were blinded to the model developer for internal and external validation of the model.

Reporting for specific materials, systems and methods

We require information from authors about some types of materials, experimental systems and methods used in many studies. Here, indicate whether each material, system or method listed is relevant to your study. If you are not sure if a list item applies to your research, read the appropriate section before selecting a response.

Materials & experimental systems

n/a	Involved in the study
☐	☒ Antibodies
☒	☐ Eukaryotic cell lines
☒	☐ Palaeontology and archaeology
☒	☐ Animals and other organisms
☒	☐ Clinical data
☒	☐ Dual use research of concern

Methods

n/a	Involved in the study
☒	☐ ChIP-seq
☒	☐ Flow cytometry
☒	☐ MRI-based neuroimaging

Antibodies

Antibodies used	The samples were incubated with antibodies against human CD3 (Pan T lymphocyte; NeoMarker, clone SP7, dilution of 1:300), CD8 (cytotoxic T lymphocytes; NeoMarker, clone SP16, dilution of 1:200), CD45RO (Memory T lymphocyte, Invitrogen, clone UCHL1, dilution of 1:400), CD66b (neutrophils; BD Pharmingen, clone G10F5, dilution of 1:200), and POSTN (Fibroblast, Abcam,ab92460, dilution of 1:200).
Validation	The antibodies were validated in previous studies (Jiang, Y., et al. ImmunoScore Signature: A Prognostic and Predictive Tool in Gastric Cancer. Ann Surg 267, 504-513 (2018).
